# Bifurcate evolution of quinone synthetases in basidiomycetes

**DOI:** 10.1186/s40694-023-00162-1

**Published:** 2023-07-03

**Authors:** Paula Sophie Seibold, Stefanie Lawrinowitz, Ihar Raztsou, Markus Gressler, Hans-Dieter Arndt, Pierre Stallforth, Dirk Hoffmeister

**Affiliations:** 1 Institute of Pharmacy, Department Pharmaceutical Microbiology, Friedrich Schiller University Jena, Winzerlaer Strasse 2, 07745 Jena, Germany; 2 Department Pharmaceutical Microbiology, Leibniz Institute for Natural Product Research and Infection Biology – Hans Knöll Institute, Winzerlaer Strasse 2, 07745 Jena, Germany; 3 Department Paleobiotechnology, Leibniz Institute for Natural Product Research and Infection Biology – Hans Knöll Institute, Winzerlaer Strasse 2, 07745 Jena, Germany; 4 Institute of Organic Chemistry and Macromolecular Chemistry, Friedrich-Schiller-Universität Jena, Humboldtstrasse 10, 07743 Jena, Germany

**Keywords:** Atromentin, Basidiomycota, *Hapalopilus*, Natural products, Polyporic acid, Quinone synthetase, *Psilocybe*, *Terana*

## Abstract

**Background:**

The terphenylquinones represent an ecologically remarkable class of basidiomycete natural products as they serve as central precursors of pigments and compounds that impact on microbial consortia by modulating bacterial biofilms and motility. This study addressed the phylogenetic origin of the quinone synthetases that assemble the key terphenylquinones polyporic acid and atromentin.

**Results:**

The activity of the *Hapalopilus rutilans* synthetases HapA1, HapA2 and of *Psilocybe cubensis* PpaA1 were reconstituted in Aspergilli. Liquid chromatography and mass spectrometry of the culture extracts identified all three enzymes as polyporic acid synthetases. PpaA1 is unique in that it features a C-terminal, yet catalytically inactive dioxygenase domain. Combined with bioinformatics to reconstruct the phylogeny, our results demonstrate that basidiomycete polyporic acid and atromentin synthetases evolved independently, although they share an identical catalytic mechanism and release structurally very closely related products. A targeted amino acid replacement in the substrate binding pocket of the adenylation domains resulted in bifunctional synthetases producing both polyporic acid and atromentin.

**Conclusions:**

Our results imply that quinone synthetases evolved twice independently in basidiomycetes, depending on the aromatic α-keto acid substrate. Furthermore, key amino acid residues for substrate specificity were identified and changed which led to a relaxed substrate profile. Therefore, our work lays the foundation for future targeted enzyme engineering.

**Supplementary Information:**

The online version contains supplementary material available at 10.1186/s40694-023-00162-1.

## Introduction

The terphenylquinones are among the most prominent mushroom natural products: the intense colors familiar from fruiting bodies of boletes or bracket mushrooms is conferred by members of this class of natural products or their direct derivatives [1]. Furthermore, phenomena such as the instantly developing cobalt blue hue that occurs after bruising of e.g., a Cornflower Bolete (*Gyroporus cyanescens*) [2] contribute to an enigmatic aura associated with these mushrooms. However, the true relevance of terphenylquinones and their derivatives reaches far beyond a hue or a blueing reaction: Prokaryotic as well as eukaryotic members of microbial consortia synthesize natural products to communicate and cooperate, or for defense [3–7]. In the case of the terphenylquinones, microbial consortia are impacted as these compounds inhibit bacterial motility and modulate biofilms [8]. As cell wall damage by microbial lytic enzymes was shown to elicit the cellular biosynthesis of terphenylquinones and their bioactive follow-up products which then leads to effects on bacteria, a much more ecologically complex scenario emerges how these compounds help shape microbial consortia surrounding fungal hyphae [9].

**Fig. 1:**
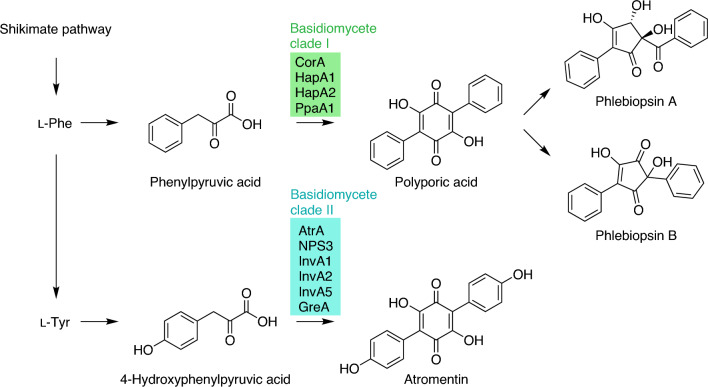
Chemical structures of basidiomycete natural products, assembled by clade I or clade II quinone synthetases, and their respective aromatic α-keto acid substrates. The conversion of polyporic acid into phlebiopsins is observed with various fungi

**Fig. 2:**
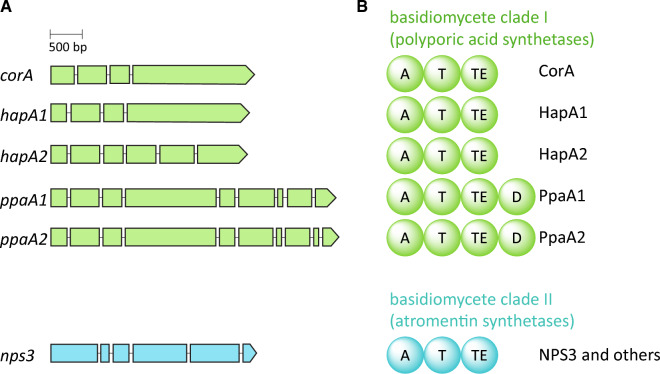
Basidiomycete quinone synthetase genes and enzymes. **A** Intron-/exon structure of the *Terana* *caerulea* gene *corA*, *Hapalopilus rutilans* genes *hapA1* and *hapA2* and *Psilocybe cubensis* genes *ppaA1* and *ppaA2*. Lines represent introns, boxes exons. **B** Domain setup of quinone synthetases. Domain acronyms: *A* adenylation, *T* thiolation, *TE* thioesterase, *D* dioxygenase

The most important representatives of terphenylquinones are polyporic acid and atromentin (Fig. 1). Their biosyntheses follow an identical chemical logic, which is deamination of an aromatic amino acid (l-phenylalanine or l-tyrosine, respectively) into the corresponding α-keto acid which then undergoes symmetric dimerization by two consecutive Claisen-type condensations to yield the quinone [10]. Quinone synthetases have been characterized from a variety of atromentin producers, such as NPS3 from the “House Eater”, the Dry Rot fungus *Serpula lacrymans*, or AtrA from the Stalkless Paxillus *Tapinella panuoides* [8, 10–12]. The hitherto investigated representatives of quinone synthetases consistently follow a tri-domain layout, reminiscent of domains of nonribosomal peptide synthetases (NRPSs), and are composed of an adenylation (A), a thiolation (T) and a thioesterase (TE) domain (Fig. 2). The most recently characterized member of basidiomycete quinone synthetases is CorA [13]. It produces polyporic acid that feeds corticin biosynthesis in the Cobalt Crust Mushroom *Terana caerulea*. Curiously, the phylogenetic placement of CorA indicates a position separate from the previously characterized basidiomycete atromentin synthetases [13]. Although a misinterpreted phylogeny could not be ruled out initially, we hypothesized that the true evolution of basidiomycete quinone synthetases may have been more complex than expected.

Here, we report on the functional characterization of three mushroom polyporic acid synthetases, i.e., HapA1 and HapA2 of the Cinnamon Bracket Mushroom *Hapalopilus rutilans* (Polyporales) as well as PpaA1 of the “Magic Mushroom” *Psilocybe cubensis* (Agaricales). Together with CorA, they form a previously unrecognized clade (clade I) of basidiomycete quinone synthetases that is more closely related to ascomycete quinone synthetases than to basidiomycete atromentin synthetases (clade II). Our results show that the capacity of mushrooms to biosynthesize terphenylquinones evolved in parallel processes, depending on the involved substrate. Furthermore, our results may imply an outstanding ecological importance of terphenylquinones which conferred the selective pressure to evolve twice the biosynthetic capacity for this class of compounds.

## Results

### Identification and phylogenetic classification of putative quinone synthetase genes

With a content of up to 40% of the dry biomass, *H. rutilans* is a well-known producer of polyporic acid [14, 15] and, thus, represents an ideal model for more profound insight into the production of this terphenylquinone by basidiomycetes. The genome of *H. rutilans* CBS 490.95 was sequenced, yielding 34.8 Mbp of sequence data, distributed on 375 contigs. To bioinformatically browse the data for genes encoding tri-domain NRPS-like enzymes, the *corA* gene (GenBank: OM515349.1 [13]) of *Terana caerulea* served as a query. Two genetic loci, hereafter referred to as *hapA1* and *hapA2*, were identified that met the criteria: *hapA1* is 3090 bp long, disrupted by three predicted introns and encodes a 106.8 kDa protein with 964 amino acids. Similarly, *hapA2* consists of 3,054 bp with five predicted introns and encodes a 925 aa protein with a mass of 101.7 kDa. Either deduced protein shows a predicted domain arrangement A-T-TE (Fig. 2), and HapA1 and HapA2 share 68% identical and 79% similar amino acids. The amino acid sequence of CorA is 64 (59)% identical and 76 (73)% similar to that of HapA1 (HapA2) (Additional file 1: Table S1).

**Fig. 3:**
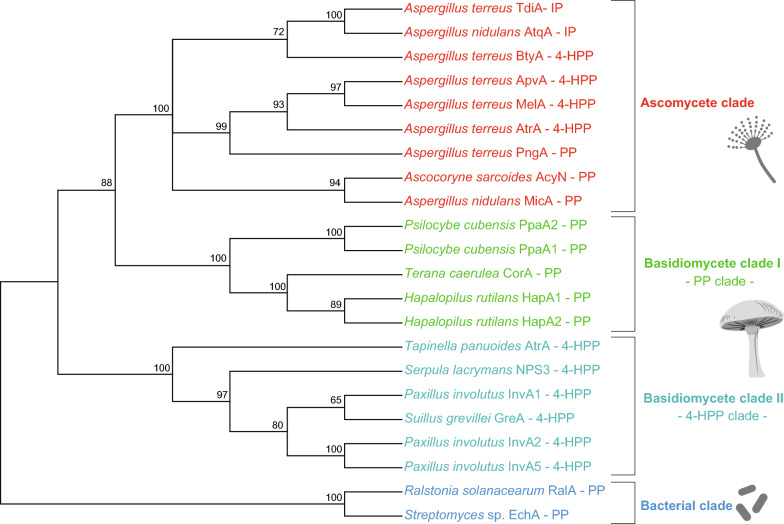
Phylogenetic analysis of quinone synthetases based on the respective adenylation domains. The A domains were aligned using ClustalW2 [69] implemented in the MEGA X software [68]. The evolutionary history was inferred by using the Maximum Likelihood method and Le_Gascuel_2008 model [70]. The percentage of replicate trees in which the associated taxa clustered together in the bootstrap test (1000 replicates) are shown next to the branches [72]. The bacterial counterparts RalA and EchA served as outgroup. Substrate acronyms: *4-HPP* 4-hydroxyphenylpyruvic acid, *IP* indole-3-pyruvic acid, *PP* phenylpyruvic acid. Please refer to the supplement for further description of the phylogenetic analysis

Surprisingly, a phylogenetic analysis of A domains of fungal tri-domain NRPS-like quinone synthetases revealed that the polyporic acid synthetase CorA from *T*. *caerulea* and the putative quinone synthetases HapA1/2 from *H. rutilans* do not cluster with basidiomycete atromentin synthetases (basidiomycete clade II, Fig. 3), even though they share an identical domain layout and yield similar terphenyl products. Rather, these enzymes form a distinct phylogenetic group (basidiomycete clade I, Fig. 3, Additional file 1: Table S2) and comprise A domains that are evolutionarily closely related to ascomycete A domains of NRPS-like enzymes. The ten-residue A domain specificity code (“NRPS code”) for HapA1/2 and related NRPSs was determined by alignment of multiple sequences of experimentally characterized other A domains, including the GrsA A domain (UniProtKB/Swiss-Prot: P0C062.1 [16, 17]) as reference (Additional file 2: Fig. S1). Valine at the first position of the code indicates α-keto acid-activating enzymes [18].

### HapA1 and HapA2 catalyze polyporic acid biosynthesis

**Fig. 4:**
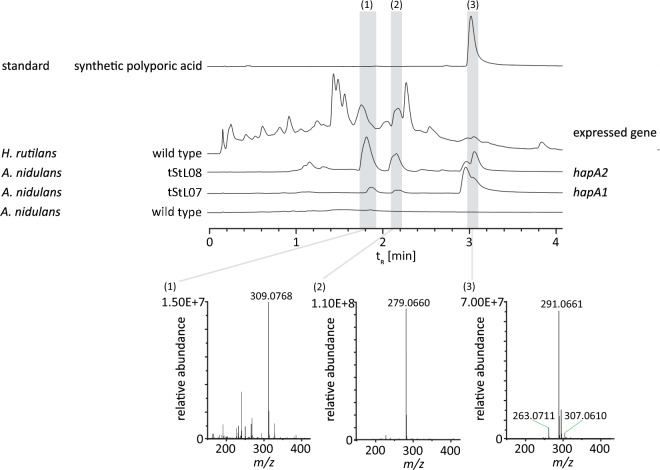
HPLC–MS HapA1 and HapA2. UHPLC profiles of metabolic extracts from cultures of *hapA1*- and *hapA2*-expressing *Aspergillus nidulans* strains tStL07 and tStL08, the untransformed *A. nidulans* wild type strain, *Hapalopilus rutilans* mycelium as well as of polyporic acid standard. Analyzed peaks are indicated by numbered bars shaded in grey. Chromatograms were extracted at λ = 350 nm. Mass spectra were recorded in negative mode. Please refer to Additional file 2: Fig. S4 for UV/Vis spectra and Additional file 1: Table S3 and Additional file 2: Fig. S3 for MS/MS data. Compound numbering: phlebiopsin A (**1**); phlebiopsin B (**2**); polyporic acid (**3**)

The cloned genes *hapA1* and *hapA2* were used to heterologously produce the proteins HapA1 and HapA2 as native, tag-less proteins in *A. nidulans* tStL07 and tStL08, respectively. The expression strains harbored the chromosomal copies integrated into the host genome under control of the alcohol-inducible promoter P*alcA*. Full length integration of the genes of interest was verified by PCR (Additional file 2: Fig. S2). The crude ethyl acetate extracts of the broth of ethanol-induced cultures of *A. nidulans* tStL07 and tStL08 were analyzed by ultra-high performance liquid chromatography/mass spectrometry (UHPLC-MS). The chromatogram showed a new signal at t_R_ = 3.0 min with a corresponding mass of *m*/*z* 291 [M-H]^−^, that was absent in control extracts of the wild type host *A. nidulans* FGSC A4 (Fig. 4). High-resolution tandem mass spectrometry (HR-MS/MS) data (Additional file 1: Table S3 and Additional file 2: Fig. S3) and the comparison with a synthetic standard and literature values [19, 20] confirmed that the new compound was identical to polyporic acid. We therefore describe HapA1 and HapA2 as polyporic acid synthetases and, together with CorA, first representatives of a new clade of quinone synthetases.

The signal at t_R_ = 3.0 min did not appear to result from a single, pure compound as two additional peaks with corresponding masses *m*/*z* 263 and 307 [M-H]^−^ and eluting at nearly the same time, were also detected. Hence, the UV/Vis spectrum (Additional file 2: Fig. S4) differs slightly from that of the synthetic polyporic acid standard. Additional signals at t_R_ = 1.8 min and 2.1 min with apparent molecular weights of *m/z* 309 and 279 [M-H]^−^, respectively, were detected as well (Fig. 4). Interestingly, these masses are compatible with those of phlebiopsin A and B, these are follow-up products of polyporic acid previously described by Kälvö et al. from the mushroom *Phlebiopsis gigantea* [21]. The masses *m/z* 263.071 and 307.061 [M-H]^−^ match those of the polyporic acid-derived intermediate pathway precursors of phlebiopsin A and B (Fig. 4 and Additional file 2: Fig. S5) and, if confirmed in our case, would support the proposed biosynthetic route [21].

### Characterization of the *P*. *cubensis* PpaA1 and PpaA2

Both *T. caerulea* and *H. rutilans*, the respective source species of CorA and HapA1/A2, belong to the Polyporales, while all hitherto characterized basidiomycete clade II (i.e., atromentin-making) synthetases originate from species of the *Boletales* [8, 10–12]. However, quinone synthetases show a taxonomic distribution beyond these two basidiomycete orders: representatives (referred to as PpaA1 and PpaA2, 80.4% identical and 90.5% similar aa, Additional file 1: Table S1) are also encoded in *Psilocybe* spp. which belong to the Agaricales [22]. The genes *ppaA1* and *ppaA2* are 4432 bp and 4471 bp long, with eight and nine predicted introns, respectively (Fig. 2). The predicted encoded proteins comprise 1333 and 1326 aa and show calculated molecular masses of 148.1 kDa (PpaA1) and 147.2 kDa (PpaA2).

Phylogenetically, the A domains of these predicted *Psilocybe* enzymes fall into clade I, i.e., the polyporic acid-clade of basidiomycete quinone synthetases (Fig. 3), yet this hypothesized enzymatic activity does not match any of the known *Psilocybe* metabolites. PpaA1 and PpaA2 both comprise the A-T-TE tri-domain familiar from both CorA, HapA1/A2 and all atromentin synthetases, but are extended by an additional C-terminal putative Fe(II)- and α-ketoglutarate-dependent dioxygenase (D) domain, according to a bioinformatic analysis using Phyre2 [23]. A potential dioxygenase activity was also supported by alignments with protein sequences of *Neurospora crassa* T7H (PDB: 5C3Q) and *Talaromyces stipitatus* TropC (PDB: 6XJJ) dioxygenases (Additional file 2: Fig. S6). A conserved iron-binding motif (HxD(x)_n_H) was identified as well as various conserved residues that may interact with α-ketoglutarate [24, 25]. The two aromatic residues F292 and Y217 were recognized as crucial for substrate binding in the T7H dioxygenase [26] and they align with F213 and F284 in TropC [27]. However, the corresponding residues P1189 and V1260 in PpaA1 make substrate binding little likely (Additional file 2: Fig. S6). A qRT-PCR analysis showed that the genes encoding PpaA1 and PpaA2 are transcribed (Additional file 2: Fig. S7). However, only marginal transcriptional regulation was found in fruiting bodies when compared with vegetative mycelium (log2-fold: 0.9 for *ppaA1* and -1.4 for *ppaA2*). These findings are somewhat consistent with published transcriptomic data of *P. cubensis* [28] that found *ppaA1* and *ppaA2* slightly upregulated in fruiting bodies versus mycelium.

### Chemical analysis of *P*. *cubensis*

Given the expressed *ppaA1* and *ppaA2* genes that may eventually lead to compound formation, we analyzed the natural products in *P. cubensis* fruiting bodies and submerse-grown mycelium by UHPLC-MS yet did not detect any traces of polyporic acid. To verify this finding by stable isotope labeling, l-[3-^13^C]phenylalanine was injected into fruiting bodies. However, signals with an apparent molecular weight of single or double ^13^C-labeled polyporic acid (*m*/*z* 292 or 293 [M-H]^−^) were not detected either.

### Characterization of PpaA1 in vivo

**Fig. 5:**
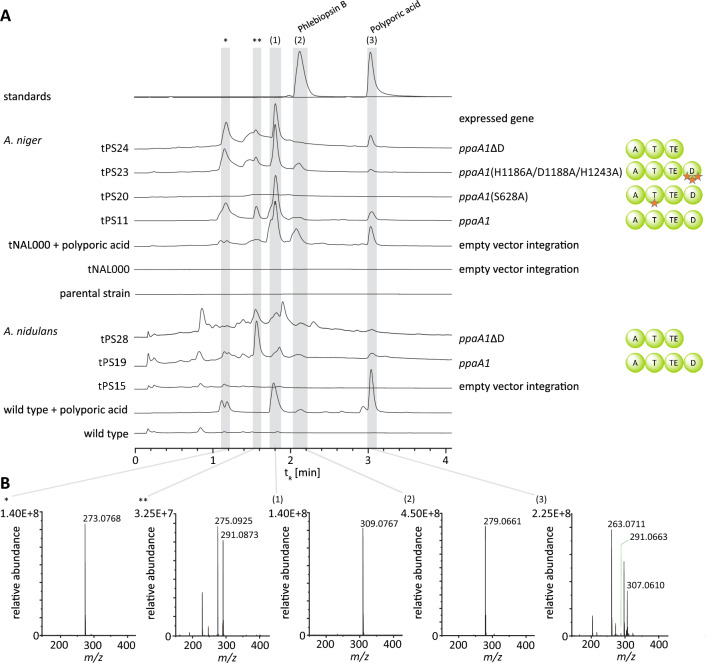
Product formation by wild type and mutated *Psilocybe cubensis* quinone synthetase PpaA1, analyzed by UHPLC-MS. Analyzed peaks are indicated by numbered bars shaded in grey. Chromatograms were extracted at λ = 350 nm. **A** Upper panel: chromatograms of synthetic polyporic acid (**3**) and of authentic phlebiopsin B (**2**). Center panel: chromatograms of ethyl acetate extracts of the culture broth of *Aspergillus niger* expressing *ppaA1* or mutated *ppaA1* variants. Bottom panel: chromatograms of ethyl acetate extracts of the culture broth of *Aspergillus nidulans* expressing *ppaA1* or mutated *ppaA1* variant. For control, extracts of untransformed parental strains and empty vector control strains *A. niger* tNAL000 and *A. nidulans* tPS15 are shown. The background conversion of added polyporic acid to phlebiopsins (phlebiopsin A (**1**)) by the latter two control strains is shown as well. **B** Mass spectra of chromatographic signals of the tPS11 extract, recorded in negative mode. UV/Vis spectra are shown in Additional file 2: Fig. S4, HR-MS and MS/MS data in Additional file 1: Table S3, Additional file 2: Figs. S11 and S13. Additional chromatograms are shown in Additional file 2: Fig. S12

As chemical analysis did not support quinone synthetase activity of PpaA1 and PpaA2, we resorted to a heterologous in vivo characterization of these yet elusive four-domain enzymes. The cDNAs of *ppaA1* and *ppaA2* were cloned into expression vector pSMX2-URA [29–31] to yield plasmids pPS14 and pPS13 (Additional file 1: Table S4) for doxycycline-inducible transgene expression in *A. niger* ATNT16Δ*pyrG*x24 [32]. These plasmids were used to create *A. niger* strains tPS11 and tPS10 (Additional file 2: Figs. S8 and S9). Transformation of plasmids pPS36 and pPS35, designed for ethanol-inducible expression of *ppaA1* and *ppaA2* in *A. nidulans* FGSC A4, led to strains tPS19 and tPS18 (Additional file 2: Fig. S9 and S10). The full-length integration of the genes was verified by PCR. The culture broths of induced transformants tPS10, tPS11, empty vector control *A. niger* tNAL000 and the untransformed *A. niger* parental strain were extracted with ethyl acetate. Subsequent UHPLC-MS analysis at λ = 350 nm revealed an additional peak at t_R_ = 3.0 min in the extract of tPS11 whose mass *m*/*z* 291 [M-H]^−^ matched that of synthetic polyporic acid (Fig. 5). Analogous results were found for *A. nidulans* tPS19 in comparison with empty vector control strain tPS15 and the untransformed host *A. nidulans*. HR-ESIMS and MS/MS-analysis showed production of polyporic acid with a corresponding mass *m*/*z* 291.066 [M-H]^−^ (Additional file 1: Table S3 and Additional file 2: Fig. S11). Unlike with *ppaA1*, heterologous expression of the *ppaA2* gene did not lead to product formation (Additional file 2: Fig. S12). However, the phenomenon of multiple (yet possibly non-functional) alleles of biosynthetic genes in basidiomycete genomes was observed previously [12, 33]. These experimental results prove polyporic acid synthetase activity for PpaA1 and, at the same time, corroborate clade I (Fig. 3) as a distinct clade of basidiomycete quinone synthetases.

**Fig. 6:**
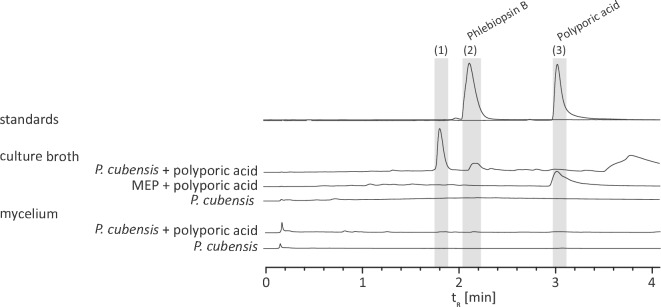
UHPLC analysis of ethyl acetate extracts of the broth and the mycelium of submerse *P. cubensis* cultures in malt extract peptone (MEP) medium, amended with polyporic acid (**3**). Control cultures were left unsupplemented or uninoculated. Analyzed peaks are indicated by numbered bars shaded in grey. Chromatograms were extracted at λ = 350 nm. Compound numbering: phlebiopsin A (**1**); phlebiopsin B (**2**)

A discrepancy remained as the chemical analysis of *Psilocybe* mycelia and mushrooms did not point to any polyporic acid in the fungal biomass (Additional file 2: Fig. S12). However, we could not rule out that this terphenylquinone is not the final product and may have been modified by downstream-acting enzymes. Therefore, submerse-grown *P. cubensis* cultures were amended with 0.5 mM polyporic acid. Besides polyporic acid, other compounds were detected (Fig. 6) which were absent in controls and which supports a scenario of polyporic acid representing an intermediate that is converted by *Psilocybe* cells. The new compounds eluted at the same retention time (t_R_ = 1.8 min and 2.1 min) as the detected substances from the *H. rutilans* and *A. nidulans* tStL07 and tStL08 ethyl acetate extracts that are described in the HapA1 and HapA2 section above. Likewise, the signals corresponded to the same masses (*m*/*z* 309 and 279 [M-H]^−^, respectively). This particular conversion also occurred in induced and, hence, *ppaA1*-expressing and polyporic acid-producing *A. niger* tPS11 and *A. nidulans* tPS19 cultures. UV/Vis spectra (Additional file 2: Fig. S4) and HR-MS/MS data (Additional file 1: Table S3, Additional file 2: Figs. S11 and S13) were in agreement with published literature values [13, 21].

Furthermore, the masses (*m*/*z* 263.071 and 307.061 [M-H]^−^) of the proposed phlebiopsin intermediates (Fig. 5 and Additional file 2: Fig. S5) were detected as well [21]. For these and for phlebiopsins A and B, increased masses by two mass units were observed when the *A. niger* tPS11 culture was supplemented with l-[3-^13^C]phenylalanine, but not when supplemented with l-[3,5-D_2_]tyrosine. For final evidence, the compound eluting at t_R_ = 2.1 min (*m*/*z* 279.066 [M-H]^−^), produced by *A. niger* tPS11, was purified by flash chromatography and semipreparative liquid chromatography. HR-MS/MS analyses (Additional file 1: Table S3 and Additional file 2: Fig. S13) and nuclear magnetic resonance (NMR) spectroscopy (Additional file 1: Table S5, Additional file 2: Figs. S14 and S15) confirmed that it is identical to phlebiopsin B.

Our findings demonstrate that *H. rutilans* HapA1 and HapA2 as well as *P. cubensis* PpaA1 are clade I polyporic acid-making quinone synthases and that these species possess the metabolic capacity to extend this pathway to phlebiopsins A and B. Given the unique domain composition of PpaA1, the question remained if the D domain was functionally involved to make phlebiopsins or if in vivo turnover occurs through an unspecific and quinone synthetase-independent reaction broadly distributed among fungi. The confirmed phlebiopsin producer *Phlebiopsis gigantea* encodes two clade I putative polyporic acid synthases (GenBank: AZAG00000000.1 [34], Additional file 2: Fig. S16) which follow the canonical A-T-TE tri-domain architecture as found with HapA1 and HapA2. This finding made a function of the PpaA1 D domain for phlebiopsin biosynthesis less likely and rather suggested a scenario that unspecific oxidoreductases and monooxygenases may catalyze this pathway extension. For more insight, a series of *ppaA1* versions was created by mutating codons within the portion encoding the iron-binding motif within the dioxygenase domain (Additional file 2: Fig. S6).

Individual amino acid exchanges included Asp1188Ala and His1243Ala, along with a triple exchange His1186Ala/Asp1188Ala/His1243Ala. Transformation with the respective plasmids led to *A. niger* strains tPS21, tPS22, and tPS23 (Additional file 1: Table S6, Additional file 2: Fig. S17). Furthermore, the entire portion encoding the D domain was deleted altogether, and the truncated gene was used for transformation to create *A. niger* tPS24 and *A. nidulans* tPS28 (Additional file 2: Figs. S17 and S18). For negative control, a *ppaA1* mutant encoding a Ser628Ala exchange was created to inactivate the T domain, as Ser628 is indispensable as phosphopantetheinyl acceptor during priming *apo*-PpaA1 into the functional *holo*-form [35]. This PpaA1 version was produced in *A. niger* tPS20 (Additional file 1: Table S6, Additional file 2: Fig. S17).

Neither polyporic acid nor phlebiopsins were detected in this particular control strain, yet present in extracts of *A. niger* strains expressing the *ppaA1* gene with point mutations in the D domain encoding region (*A. niger* tPS21, tPS22, tPS23 and tPS24, and *A. nidulans* tPS28, Fig. 5, Additional file 2: Fig. S12). When polyporic acid was added to liquid cultures of *A. niger* empty vector control tNAL000 or to *A. nidulans* wild type, phlebiopsins were produced as well (Fig. 5). This finding demonstrates that the fungal cells per se have the capacity to produce phlebiopsins from polyporic acid which implies a set of unspecific oxidative enzymes, accepting polyporic acid or the respective intermediates. In return, a catalytic function of the PpaA1 D domain appears even more unlikely.

### Domain swap of PpaA1 and CorA

**Fig. 7:**
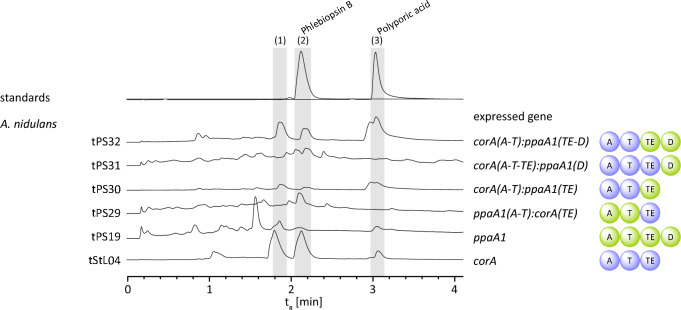
Polyporic acid formation by chimeric quinone synthetases and subsequent phlebiopsin production by the heterologous host. Shown are UHPLC-MS analyses of ethyl acetate extracts of *ppaA1:corA*-expressing *A. nidulans* strains. For comparison, chromatograms of *A. nidulans* tPS19 and tStL04, expressing native *ppaA1* and *corA*, respectively, are included. Analyzed peaks are indicated by numbered bars shaded in grey. Chromatograms were extracted at λ = 350 nm. Compound numbering: phlebiopsin A (**1**); phlebiopsin B (**2**); polyporic acid (**3**)

To clarify if the D domain, even if catalytically inactive, impacts the functionality of the other PpaA1 portions, domains were swapped between PpaA1 and CorA and the chimera investigated in vivo by expressing the respective genes in *A. nidulans* (Additional file 1: Table S6, Additional file 2: Fig. S18). Fusing the A-T PpaA1 di-domain with the CorA TE domain and the reciprocal swap in *A. nidulans* tPS29 and tPS30, respectively, led to a couple of synthetases that did not show altered catalytic activity, compared to wild type PpaA1 and CorA (produced by tPS19 and tStL04 [13]). Next, the PpaA1 D domain was C-terminally attached to CorA, while a parallel experiment included the CorA A-T di-domain joined to the TE-D di-domain of PpaA1 (*A. nidulans* tPS31 and tPS32). These chimeras did not show changed metabolic profiles either (Fig. 7) and still functioned as polyporic acid synthetases which was, in all cases, metabolized to phlebiopsin by the host.

Collectively, these results show that (i) the D domain is not essential for the production of polyporic acid by PpaA1 and (ii) that PpaA1 and CorA do not depend on their native TE domains for proper function. Furthermore, the D domain attached to CorA does not impair its catalytic activity.

### Mutation of position 5 of the nonribosomal code

**Fig. 8:**
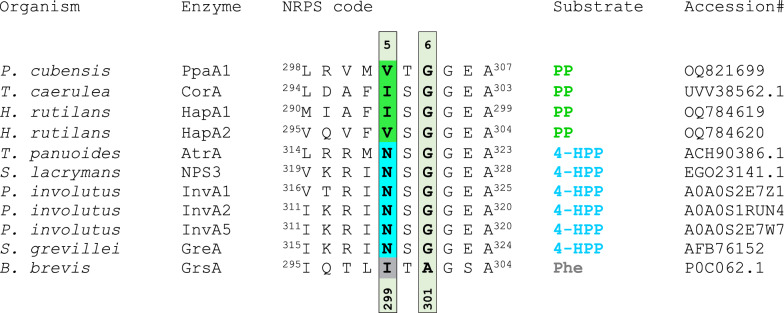
Amino acid sequences of the A domains of PpaA1, CorA, HapA1, HapA2, and related enzymes were aligned with ClustalW [69]. Specificity code positions 5 and 6 are numbered according to the GrsA sequence [16]. The amino acid on position 5 and the substrate of the respective enzyme are highlighted in matching colors. Green: Val/Ile – phenylpyruvic acid (PP); cyan: Asn – 4-hydroxyphenylpyruvic acid (4-HPP); grey: Ile – phenylalanine (Phe)

We followed up on the apparent correlation between the accepted α-keto acid substrate (phenylpyruvate vs. 4-hydroxyphenylpyruvate) and the amino acid residue at position 5 of the nonribosomal code of the adenylation domain (Fig. 8, Additional file 2: Fig. S1). This particular position correlates with the activated substrate, irrespective of an asco- or basidiomycete origin of the enzyme, and is occupied by aliphatic amino acids (Ile or Val) in polyporic acid synthetases whereas atromentin synthetases, such as NPS3 of *Serpula lacrymans*, show an asparagine. Therefore, a *corA* mutant encoding an Ile298Asn exchange, a *ppaA1* mutant encoding Val302Asn and two *nps3* mutants encoding Asn323Ile and Asn323Val exchanges, respectively, were created. Molecular modeling predicted that these amino acid positions are located in immediate proximity of the substrate (Additional file 2: Fig. S19). Additionally, wild type NPS3 was produced for control. Transformation with the respective plasmids led to *A. nidulans* strains tPS35, tPS36, tPS37, tPS38 and tPS39 (Additional file 1: Table S6, Additional file 2: Fig. S20).

**Fig. 9:**
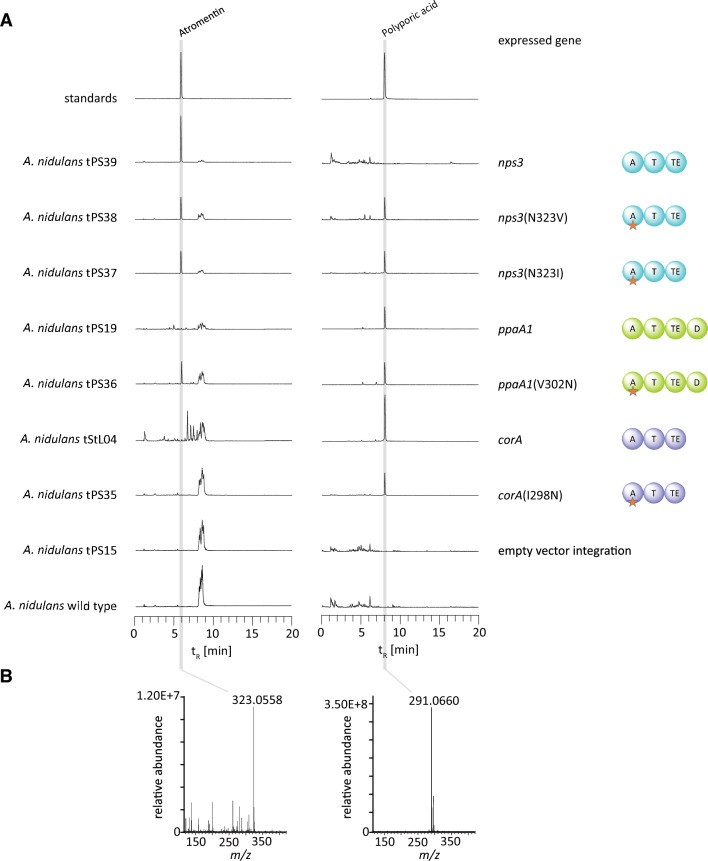
HPLC–MS analysis of mutated quinone synthetases. Shown are extracted ion chromatograms (EICs). **A** Chromatograms of polyporic acid and atromentin standards, chromatograms of ethyl acetate extracts of the culture broth of *A. nidulans* expressing *corA*, *ppaA1*, *nps3*, or mutated *corA*, *ppaA1* or *nps3* variants. As controls, extracts of the untransformed *A. nidulans* parental strain and empty vector control strain *A. nidulans* tPS15 are shown. **B** Mass spectra of chromatographic signals of the tPS36 extract, recorded in negative mode, match the calculated masses of atromentin and polyporic acid (*m*/*z* 323.0561 and *m*/*z* 291.0663 [M-H].^−^). MS/MS data are shown in Additional file 2: Fig. S21

The culture broths of the above-mentioned strains were extracted with ethyl acetate. The extracts of polyporic acid-producing strains *A. nidulans* tPS19 (expressing *ppaA1*) and tStL04 (expressing *corA* [13]) as well as the non-producing empty vector negative control strain tPS15 and *A. nidulans* wild type were analyzed by HPLC-HRESIMS and MS/MS (Fig. 9, Additional file 1: Table S3, Additional file 2: Fig. S21).

Product formation was confirmed by comparison of HR-MS data of extracts with those of standards of atromentin and polyporic acid. Confirming previous in vitro data [8] on NPS3 as an atromentin synthetase, this compound was found as the single in vivo product of tPS39 (encoding *nps3*). Interestingly, both atromentin and polyporic acid were present in extracts expression strains tPS37 and tPS38, expressing *nps3* engineered to introduce Ile and Val, respectively, on position 5. Likewise, the inverse experiment with the polyporic acid synthetase PpaA1, engineered to introduce Asn as the signature residue of atromentin synthetases, led to simultaneous production of polyporic acid and atromentin in *A. nidulans* tPS36. Surprisingly, the analogously altered CorA version*,* made by tPS35, retained strict specificity for polyporic acid. Hence, position 5 of the nonribosomal code of quinone synthetases was identified to relax and contribute to, but not strictly determine, their substrate specificity.

## Discussion

The terphenylquinones are an intriguing class of natural products. Historically, basidiomycete natural product research began with the isolation of polyporic acid and atromentin from mushroom fruiting bodies [14, 36]. Knowledge on mushroom natural product biosyntheses, accumulating since, also proved the terphenylquinones as versatile source for the pathways toward the pulvinic and thelephoric acids, grevillins, diarylcyclopentenones, xylerythrines and the badiones [1, 2, 37]. More than a century after their discovery, the ecological role of terphenylquinones and their follow-up products began to emerge. The leucomentins, i.e., esters of atromentin that release osmundalactone, exert strong insecticidal effects and protect mushroom fruiting bodies from larvae feeding on them [38]. Pulvinic acids impact upon the surrounding microbiome by inhibiting bacterial swarming and modulating biofilm formation [39]. Brown-rotting fungi use variegatic acid as a reductant for Fe^3+^ ions as hydroxyl radicals are formed by Fenton chemistry when lignocellulose is degraded [40, 41]. Therefore, multiple functions and bioactivities may have conferred an ecological advantage to terphenylquinone-producing mushrooms and our results show that the basidiomycetes evolved this biosynthetic capacity twice which underscores its importance. Curiously, simultaneous production of both polyporic acid and atromentin by one enzyme has not been reported yet. Our present discovery of two independent clades of basidiomycete quinone synthetases seems to indirectly support a strictly separated metabolism under physiological conditions. Furthermore, this study did not provide evidence for synthetases catalyzing heterodimerization of phenylpyruvate and 4-hydroxyphenylpyruvate.

However, the two quinone synthetase clades share an identical biochemistry. They adenylate aromatic α-keto acid monomers (derived from l-Phe or l-Tyr) and symmetrically dimerize these by Claisen-/Dieckmann-type condensations into terphenylquinones [2, 10]. Consequently, polyporic acid synthetases, such as HapA1, HapA2 and CorA follow the identical mechanistic logic as atromentin synthetases and feature a tri-domain setup of adenylation-, thiolation-, and thioesterase domains. Our work revealed that the non-canonical tetra-domain enzyme PpaA1 is a functional polyporic acid synthetase as well although it includes an extra dioxygenase domain. Its function warrants further investigation as a catalytic role did not become evident during this study. Prior investigations on other peptide synthetases or related enzymes demonstrated a requirement for extra domains in various cases, probably not as active catalyst, rather due to structural integrity of the other domains within the enzyme. For example, fungal α-amino adipate reductases feature an incomplete and catalytically incompetent N-terminal condensation domain whose strict requirement for the activity of the downstream adenylation domain was shown [42]. Yet, an opposite scenario seems to apply for PpaA1 as it remained fully active after the D domain had been removed.

From a systematic perspective, the Polyporales were primarily recognized as polyporic acid producers [2, 37], represented e.g., by species in the genera *Terana*, *Phanerochaete*, and *Hapalopilus*. Our work on *Psilocybe cubensis* PpaA1 demonstrates the capacity to biosynthesize this terphenylquinone for a member of the Agaricales as well. Furthermore, the results help chart the *Psilocybe* secondary metabolome. Prior work on this genus concentrated almost exclusively on the psychotropic psilocybin and related compounds, and to a degree on monoamine oxidase-inhibiting β-carbolines [43–47], i.e., metabolites that derive from l-tryptophan. In contrast, l-phenylalanine was previously not known to feed a *Psilocybe* secondary metabolic pathway. Although *ppaA1* was actively transcribed and slightly up-regulated in fruiting bodies, as yet unidentified biotic or abiotic triggers may exist that would lead to a stronger gene expression and detectable amounts of polyporic acid and/or follow-up metabolites in *Psilocybe* mushrooms. In fact, microorganisms surrounding the fungal hyphae may elicit natural product biosyntheses. Prominent examples for ascomycetes include the interaction of *Streptomyces rapamycinicus* and Aspergilli, which induced the biosynthesis of the polyketide orsellinic acid and derivatives in *A. nidulans* and fumicyclins in *A. fumigatus* [48, 49].

Similar phenomena have been observed for basidiomycetes as well: *Bacillus subtilis* triggered production of the sesquiterpene lagopodin B in *Coprinopsis cinerea* [50] and the above-mentioned quinone synthetase gene *nps3* in *Serpula lacrymans* is massively upregulated in the presence of bacteria secreting lytic enzymes [9]. Yet, interactions with other microbes and potential consequences for natural product formation are entirely unknown and still uninvestigated for *Psilocybe*. Our results on a phylogenetically dual emergence of quinone synthetases in basidiomycetes imply a more complex evolution of biosynthetic capacities for natural products and, at the same time, underline the ecological relevance of terphenylquinones and their derivatives.

**Methods** (please refer also to Additional file 3).

### Microbiological methods

*Psilocybe cubensis* FSU12407 was maintained on malt extract peptone (MEP) agar plates (per liter: 30 g malt extract, 3 g peptone, 18 g agar, pH 5.6). To collect biomass from liquid cultures, *P*. *cubensis* was cultivated for 7 days in MEP medium at 25 °C and 140 rpm. Carpophore formation was induced as described [47]. Fungal biomass was collected, washed with water if harvested from a liquid culture, shock-frozen in liquid nitrogen and lyophilized prior to nucleic acid or metabolite extraction. *Terana caerulea* CBS 452.86 was cultivated as described previously [13]. For genomic DNA isolation, *Hapalopilus rutilans* CBS 490.95 was grown in 100 mL liquid MEP medium at 25 °C, shaken at 100 rpm in the dark, for 10 days. For extraction of polyporic acid, *H. rutilans* was grown on MEP agar plates for 21 days at 25 °C. *Serpula lacrymans* S7 [40] was grown on MEP agar plates for 10 days at 25 °C. *Escherichia coli* XL1-blue was used for routine cloning and plasmid propagation. For cultivation of *E. coli*, LB medium (per liter: 5 g yeast extract, 10 g tryptone, 10 g NaCl, 18 g agar) supplemented with 100 µg mL^−1^ carbenicillin was used. *Aspergillus niger* ATNT16ΔpyrGx24 [31] and *A. nidulans* FGSC A4 were used as hosts for heterologous expression. *Aspergillus* strains were maintained on *Aspergillus* minimal medium (AMM) [51] agar plates (20 g L^−1^ agar, pH 6.5) supplemented with 50 mM d-glucose and 5 mM l-glutamine at 30 °C for 5 days (*A. niger*) or at 37 °C for 3 days (*A. nidulans*). Media for *A. niger* ATNT16ΔpyrGx24 were additionally supplemented with 10 mM uridine, media for *A. nidulans* transformants with 0.1 µg mL^−1^ pyrithiamine hydrobromide. Conidia were harvested as described previously [33]. For genetic analysis transformants and control strains were grown for 3 days in 24 well plates with 2 mL YPD (per liter: 5 g yeast extract, 20 g soy peptone, 20 g d-glucose, pH 6.5) liquid medium per well. To induce gene expression in recombinant *A. niger*, the strains were cultivated in 100 mL AMM containing 100 mM d-glucose and 20 mM l-glutamine at 30 °C, 140 rpm for 18 h, 30 µg mL^−1^ doxycycline hydrochloride was added and cultivation was continued for additional 48 h. For product formation in *A. nidulans*, the strains were cultivated in 100 mL AMM containing 5 mM d-glucose, 5 mM l-glutamine and 200 mm ethanol at 30 °C, shaken at 140 rpm for 72 h. Media were inoculated at a titer of 1∙10^6^ conidia per milliliter. For stable-isotope labeling, we injected 500 µL 10 mM l-[3-^13^C]phenylalanine (CortecNet) into *P. cubensis* fruiting bodies or added 1 mM l-[3-^13^C]phenylalanine or l-[3,5-D_2_]tyrosine into AMM liquid medium for *A. niger*. Polyporic acid feeding experiments were conducted with 0.5 mM substance in liquid MEP medium or AMM.

### Molecular biological methods

Genomic DNA from *P. cubensis*, *T. caerulea, S. lacrymans* and *Aspergillus* spp. was isolated following a described protocol with slight modifications (isopropanol instead of ethanol precipitation) [52]. For genomic sequencing of *H. rutilans*, gDNA was extracted using a modified protocol originally developed for plants [53]. Mycelium was ground under liquid nitrogen, the biomass was resuspended in 700 µL LETS buffer supplemented with 100 µL CTAB buffer (Promega), and incubated at 65 °C for 1 h. The sample was centrifuged, and the supernatant was extracted with phenol/chloroform/isoamylalcohol (25:24:1, v/v/v). The liquid phase was treated with 3 µL Monarch RNase A (20 mg mL^−1^, NEB) at 37 °C for 45 min, and subsequently extracted twice with phenol/chloroform/isoamylalcohol (25:24:1, v/v/v). The gDNA was precipitated by adding 0.2 M NaCl and 3 volumes cold 95% (v/v) ethanol to the liquid phase, followed by incubation at −20 °C for 1 h, and washed ten times with ice-cold 70% (v/v) ethanol. The DNA pellet was dissolved in 10 mM TRIS buffer, pH 8.0. RNA isolation, reverse transcription, and qRT-PCR were performed as described [54–56]. The housekeeping reference gene *enoA*, encoding enolase, served as internal standard. Oligonucleotides used for qRT-PCR are listed in Additional file 1: Table S7. Gene expression levels were determined as described [57]. PEG-mediated protoplast transformation of *A. niger* and *A. nidulans* was carried out as previously described [31]. Full length integration was confirmed by PCR (Additional file 1: Table S8; PCR methods A, B and C). The transformants are shown in Table S6. Oligonucleotides used for cloning, colony PCRs and DNA sequencing are listed in Additional file 1: Table S9.

### Genome sequencing and bioinformatic analysis

Genomic DNA sequencing of *H. rutilans* was carried out using an Oxford Nanopore MinION flow cell. The genome was assembled using CANU (version 2.1) [58, 59] based on an expected genome size of 50 Mbp. Signal level reads were indexed against the draft genome using Nanopolish software [60, 61]. After using minimap [62] and samtools [63, 64] to sort and map the reads, a consensus sequence was calculated using Nanopolish [65]. Sequence analysis and alignments were conducted with Geneious software (version 7.1.9). Gene and coding sequence prediction was carried out using Augustus (version 0.1.1) [66] and compared with BLAST [67].

Phylogenetic analyses were conducted using MEGA X [68] based on the sequences of A domains (Fig. 3, Additional file 1: Table S2, Additional file 2: Fig. S16). Protein sequences were aligned using ClustalW2 [69]. The evolutionary history was inferred by using the Maximum Likelihood method and Le_Gascuel_2008 model [70]. A phylogenetic tree was constructed using the Maximum Likelihood method and the Jones-Taylor-Thornton model [71] and 1000 bootstrap replications [72].

Quinone synthetase modeling was performed with AlphaFold2 [73] and was superimposed using ChimeraX [74, 75] and McyG (PDBe 4r0m, [76]) as reference (Additional file 2: Fig. S19).

### Chemical synthesis of polyporic acid

Polyporic acid (2,5-dihydroxy-3,6-diphenyl-1,4-benzoquinone) was synthesized in an analogous manner as described in [77] and [78]. The full procedure is described in Additional file 3: Experimental procedures. NMR data is shown in Additional file 2: Figs. S22 and S23.

### Analytical and preparative methods

To analyze natural products, mycelium of *H. rutilans* was harvested from MEP agar plates, *P*. *cubensis* mycelium was harvested from liquid cultures, and carpophores were collected, lyophilized and ground under liquid nitrogen to a fine powder. 1 mL water per 10 mg dry biomass was added and acidified with HCl to pH ≤ 2. The culture filtrates of *A. niger* and *A. nidulans* transformants and control strains were also acidified. The samples were extracted with the same volume of ethyl acetate. The organic phase was collected, dried over anhydrous sodium sulfate, and evaporated to dryness. The crude extracts were dissolved in MeOH and subjected to UHPLC-MS analysis on an Agilent 1290 Infinity II instrument, interfaced to an Agilent 6130 single quadrupole mass detector, operated in alternating positive/negative mode and applying solvent gradient A (Additional file 1: Table S10). UV/Vis spectra were recorded between λ = 200–600 nm and chromatograms were extracted at λ = 350 nm. To purify phlebiopsin B from fungal cultures for NMR spectroscopy, *A. niger* tPS11 was grown in a 10 L culture, dispensed in 2 L Erlenmeyer flasks, under the conditions described in the Microbiological Methods section above. The filtrate was extracted with ethyl acetate as described above. The dried crude extract was then dissolved in 50 mL CH_2_Cl_2_, centrifuged (10 min, 11,000 × g), and the supernatant was loaded onto a 40 g Flash Pure Silica column of a Büchi C810 Flash chromatograph. The normal phase purification was carried out with CH_2_Cl_2_ and MeOH, using gradient B (Additional file 1: Table S10). UV/Vis spectra were recorded between λ = 200–400 nm and chromatograms were extracted at λ = 254, 280, 320, and 350 nm. The fraction with the highest absorbance at 350 nm was collected, dried, dissolved in 1.5 mL MeOH, centrifuged (10 min, 11,000 × g), and the supernatant was injected into a 12 g C18 Column in the same Flash chromatograph. The reverse phase purification was accomplished using gradient C (Additional file 1: Table S10). The fraction with the highest absorbance at 350 nm was collected, dried, solved in 1 mL MeOH and subjected to reversed phase semi-preparative HPLC, using method D (Additional file 1: Table S10) with an Agilent Eclipse XDB C18 column (9.4 × 250 mm, 5 µm particle size, thermostatted at 30 °C). High resolution mass spectra and tandem MS fragmentation patterns were recorded on a Thermo Scientific Exactive Orbitrap instrument (Additional file 1: Table S10, methods E and F). 1D and 2D NMR spectra of phlebiopsin B were recorded on a Bruker Avance III spectrometer operated at 600 or 150 MHz and 300 K. Solvent signals were referenced to δ_H_ = 3.31 ppm and δ_C_ = 49.0 ppm for CHD_2_OD.

## Supplementary Information

**Additional file 1.** Ten supplementary tables with supporting research data, materials and methods.

**Additional file 2.** 23 supplementary figures with supporting biological and chemical data.

**Additional file 3.** Experimental procedures.

## Data Availability

The sequences of *hapA1* and *hapA2* genes from *H. rutilans* and *ppaA1* and *ppaA2* from *P. cubensis* are deposited under the GenBank accession numbers OQ784619; OQ784620, OQ821699, and OR105898, respectively.
