# Supplementary material for: Bifurcate evolution of quinone synthetases in basidiomycetes
Source: Fungal Biol Biotechnol. 2023 Jul 3;10:14. doi: 10.1186/s40694-023-00162-1 (PMC10316625; doi:10.1186/s40694-023-00162-1)
Supplement: Supplementary file 1 — Additional file 1. Ten supplementary tables with supporting research data, materials and methods. [file 40694_2023_162_MOESM1_ESM.pdf]

Table S1. Sequence identities and similarities. Pairwise identity [%]/pairwise positive [%].

	CorA	HapA1	HapA2	PpaA1	PpaA2
**CorA**	100/100	64/76	59/73	48/65	49/66
**HapA1**		100/100	68/79	49/66	50/66
**HapA2**			100/100	47/64	47/63
**PpaA1**				100/100	80/91
**PpaA2**					100/100

Table S2. Protein sequences used for phylogenetic analysis of quinone synthetases. Substrates are: IP, indole-3-pyruvate; PP, phenylpyruvate; 4-HPP, 4-hydroxyphenylpyruvate.

**Organism**	**Enzyme**	**Amino acids**	**Substrate**	**Direct or pathway product**	**Accession**	**Reference**
Basidiomycota						
Psilocybe cubensis	PpaA1	1333	PP	polyporic acid	OQ821699	This work
Psilocybe cubensis	PpaA2	1326	PP	none detected	OR105898	This work
Terana caerulea	CorA	994	PP	polyporic acid	UVV38562.1	[1]
Hapalopilus rutilans	HapA1	964	PP	polyporic acid	OQ784619	This work
Hapalopilus rutilans	HapA2	925	PP	polyporic acid	OQ784620	This work
Tapinella panuoides	AtrA	957	4-HPP	atromentin	ACH90386.1	[2]
Serpula lacrymans	Nps3	962	4-HPP	atromentin	EGO23141.1	[3]
Paxillus involutus	InvA1	959	4-HPP	atromentin	A0A0S2E7Z1	[4]
Paxillus involutus	InvA2	953	4-HPP	atromentin	A0A0S1RUN4	[4]
Paxillus involutus	InvA5	953	4-HPP	atromentin	A0A0S2E7W7	[4]
Suillus grevillei	GreA	958	4-HPP	atromentin	AFB76152	[5]
Ascomycota						
Aspergillus nidulans	MicA	938	PP	microperfuranone	Q5B7T4	[6]
Ascocoryne sarcoides	AcyN	930	PP	polyporic acid	P9WES4.1	[7]
Aspergillus terreus	PngA	946	PP	phenguignardic acid	Q0CBN5	[8]
Aspergillus terreus	AtrA	920	4-HPP	atromentin	Q0CT94	[9}
Aspergillus terreus	MelA	925	4-HPP	aspulvinone E	A0A336U965	[10]
Aspergillus terreus	ApvA	925	4-HPP	aspulvinone E	Q0CWD0	[11]
Aspergillus terreus	BtyA	930	4-HPP	butyrolactone II	Q0CU19	[11]
Aspergillus terreus	AtqA	962	IP	didemethylasterriquinone D	Q0D034	[11]
Aspergillus nidulans	TdiA	949	IP	didemethylasterriquinone D	CBF80711.1	[12]
Bacteria						
Streptomyces sp.	EchA	967	PP	echoside	AHN91924	[13]
Ralstonia solanacearum	RalA	937	PP	ralfuranone B	AEC03968.1	[14]

References

1. Lawrinowitz S, Wurlitzer JM, Weiss D, Arndt HD, Kothe E, Gressler M, Hoffmeister D: **Blue Light-Dependent Pre-mRNA Splicing Controls Pigment Biosynthesis in the Mushroom *Terana caerulea***. *Microbiol Spectr* 2022, **10**(5):e0106522.
2. Schneider P, Bouhired S, Hoffmeister D: **Characterization of the atromentin biosynthesis genes and enzymes in the homobasidiomycete *Tapinella panuoides***. *Fungal Genet Biol* 2008, **45**(11):1487-1496.

3. Tauber JP, Schroeckh V, Shelest E, Brakhage AA, Hoffmeister D: **Bacteria induce pigment formation in the basidiomycete *Serpula lacrymans***. *Environ Microbiol* 2016, **18**(12):5218-5227.
4. Braesel J, Götze S, Shah F, Heine D, Tauber J, Hertweck C, Tunlid A, Stallforth P, Hoffmeister D: **Three Redundant Synthetases Secure Redox-Active Pigment Production in the Basidiomycete *Paxillus involutus***. *Chem Biol* 2015, **22**(10):1325-1334.
5. Wackler B, Lackner G, Chooi YH, Hoffmeister D: **Characterization of the *Suillus grevillei* quinone synthetase GreA supports a nonribosomal code for aromatic α-keto acids**. *ChemBioChem* 2012, **13**(12):1798-1804.
6. Yeh HH, Chiang YM, Entwistle R, Ahuja M, Lee KH, Bruno KS, Wu TK, Oakley BR, Wang CC: **Molecular genetic analysis reveals that a nonribosomal peptide synthetase-like (NRPS-like) gene in *Aspergillus nidulans* is responsible for microperfuranone biosynthesis**. *Appl Microbiol Biotechnol* 2012, **96**(3):739-748.
7. Wieder C, Peres da Silva R, Witts J, Jager CM, Geib E, Brock M: **Characterisation of ascocorynin biosynthesis in the purple jellydisc fungus *Ascocoryne sarcoides***. *Fungal Biol Biotechnol* 2022, **9**(1):8.
8. Sun WW, Guo CJ, Wang CCC: **Characterization of the product of a nonribosomal peptide synthetase-like (NRPS-like) gene using the doxycycline dependent Tet-on system in *Aspergillus terreus***. *Fungal Genet Biol* 2016, **89**:84-88.
9. Hühner E, Backhaus K, Kraut R, Li SM: **Production of alpha-keto carboxylic acid dimers in yeast by overexpression of NRPS-like genes from *Aspergillus terreus***. *Appl Microbiol Biotechnol* 2018, **102**(4):1663-1672.
10. Geib E, Gressler M, Viediernikova I, Hillmann F, Jacobsen ID, Nietzsche S, Hertweck C, Brock M: **A Non-canonical Melanin Biosynthesis Pathway Protects *Aspergillus terreus* Conidia from Environmental Stress**. *Cell Chem Biol* 2016, **23**(5):587-597.
11. Guo CJ, Knox BP, Sanchez JF, Chiang YM, Bruno KS, Wang CC: **Application of an efficient gene targeting system linking secondary metabolites to their biosynthetic genes in *Aspergillus terreus***. *Org Lett* 2013, **15**(14):3562-3565.
12. Schneider P, Weber M, Rosenberger K, Hoffmeister D: **A one-pot chemoenzymatic synthesis for the universal precursor of antidiabetes and antiviral bis-indolylquinones**. *Chem Biol* 2007, **14**(6):635-644.
13. Zhu J, Chen W, Li YY, Deng JJ, Zhu DY, Duan J, Liu Y, Shi GY, Xie C, Wang HX *et al*: **Identification and catalytic characterization of a nonribosomal peptide synthetase-like (NRPS-like) enzyme involved in the biosynthesis of echosides from *Streptomyces* sp. LZ35**. *Gene* 2014, **546**(2):352-358.
14. Wackler B, Schneider P, Jacobs JM, Pauly J, Allen C, Nett M, Hoffmeister D: **Ralfuranone biosynthesis in *Ralstonia solanacearum* suggests functional divergence in the quinone synthetase family of enzymes**. *Chem Biol* 2011, **18**(3):354-360.

Table S3. HR-MS and MS/MS data of polyporic acid and phlebiopsins A and B.

Compound	Formula	Neutral mass [M]	Parental ion *m/z* [M-H]$^-$	MS/MS specific ions	
				Ion mass *m/z* [M-H]$^-$	Formula*
Polyporic acid	$C_{18}H_{12}O_4$	292.0736	291.066		
				263.017	$C_{17}H_{11}O_3$
				191.086	$C_{15}H_{11}$
				117.033	C_8H_5O
Phlebiopsin A	$C_{18}H_{14}O_5$	310.0841	309.077		
				291.066	$C_{18}H_{11}O_4$
				281.081	$C_{17}H_{13}O_4$
				265.087	$C_{17}H_{13}O_3$
				251.071	$C_{16}H_{11}O_3$
				235.076	$C_{16}H_{11}O_2$
				191.086	$C_{15}H_{11}$
				187.039	$C_{11}H_7O_3$
				147.044	$C_9H_7O_2$
				119.049	C_8H_7O
				117.033	C_8H_5O
Phlebiopsin B	$C_{17}H_{12}O_4$	280.0736	279.066		
				251.071	$C_{16}H_{11}O_3$
				235.076	$C_{16}H_{11}O_2$
				191.086	$C_{15}H_{11}$
				179.086	$C_{14}H_{11}$
				121.028	$C_7H_5O_2$
				117.033	C_8H_5O
				105.033	C_7H_5O
				77.038	C_6H_5
Atromentin	$C_{18}H_{12}O_6$	324.0634	323.056		
				295.061	$C_{17}H_{11}O_5$
				251.071	$C_{16}H_{11}O_3$
				223.076	$C_{15}H_{11}O_2$
				133.028	$C_8H_5O_2$
				117.033	C_8H_5O
				105.033	C_7H_5O

*Predicted with ChemCalc [1] and CFM-ID (version 4.0) [2-6]

References

1. Patiny L, Borel A: **ChemCalc: a building block for tomorrow's chemical infrastructure**. *J Chem Inf Model* 2013, **53**(5):1223-1228.
2. Wang F, Liigand J, Tian S, Arndt D, Greiner R, Wishart DS: **CFM-ID 4.0: More Accurate ESI-MS/MS Spectral Prediction and Compound Identification**. *Anal Chem* 2021, **93**(34):11692-11700.
3. Djoumbou-Feunang Y, Pon A, Karu N, Zheng J, Li C, Arndt D, Gautam M, Allen F, Wishart DS: **CFM-ID 3.0: Significantly Improved ESI-MS/MS Prediction and Compound Identification**. *Metabolites* 2019, **9**(4).
4. Allen F, Pon A, Greiner R, Wishart D: **Computational Prediction of Electron Ionization Mass Spectra to Assist in GC/MS Compound Identification**. *Anal Chem* 2016, **88**(15):7689-7697.
5. Allen F, Greiner R, Wishart D: **Competitive fragmentation modeling of ESI-MS/MS spectra for putative metabolite identification**. *Metabolomics* 2015, **11**:98-110.
6. Allen F, Pon A, Wilson M, Greiner R, Wishart D: **CFM-ID: a web server for annotation, spectrum prediction and metabolite identification from tandem mass spectra**. *Nucleic Acids Res* 2014, **42**(Web Server issue):W94-99.

Table S4. Plasmids.

Plasmid name	Vector backbone	Gene product	Position of His$_6$-tag	Reference
pSMX2-URA	pUC19	- (vector)	-	[1]
pMG49	pMD03	LpaA	-	[2]
pPS13	pSMX2-URA	PpaA2	N-terminus	this study
pPS14	pSMX2-URA	PpaA1	N-terminus	this study
pPS29	pMG49	- (vector)	-	this study
pPS35	pPS29	PpaA2	C-terminus	this study
pPS36	pPS29	PpaA1	C-terminus	this study
pPS37	pSMX2-URA	PpaA1^{S628A}	N-terminus	this study
pPS39	pSMX2-URA	PpaA1^{D1188A}	N-terminus	this study
pPS40	pSMX2-URA	PpaA1^{H1243A}	N-terminus	this study
pPS41	pSMX2-URA	PpaA1$^{H1186A/D1188A/H1243A}$	N-terminus	this study
pPS42	pSMX2-URA	PpaA1ΔD-domain	N-terminus	this study
pPS50	pPS29	PpaA1ΔD-domain	C-terminus	this study
pPS51	pPS29	PpaA1(A-T)::CorA(TE)	C-terminus	this study
pPS52	pPS29	CorA(A-T)::PpaA1(TE)	C-terminus	this study
pPS53	pPS29	CorA(A-T-TE)::PpaA1(D)	C-terminus	this study
pPS54	pPS29	CorA(A-T)::PpaA1(TE-D)	C-terminus	this study
pPS57	pPS29	CorA^{I298N}	C-terminus	this study
pPS58	pPS29	PpaA1^{V302N}	C-terminus	this study
pPS59	pPS29	Nps3^{N323I}	C-terminus	this study
pPS60	pPS29	Nps3^{N323V}	C-terminus	this study
pPS61	pPS29	Nps3	C-terminus	this study
pSS06	pMG49	CorA	-	[3]
pSS10	pMG49	HapA1	-	this study
pSS11	pMG49	HapA2	-	this study

References

1. Geib E, Brock M: **ATNT: an enhanced system for expression of polycistronic secondary metabolite gene clusters in *Aspergillus niger***. *Fungal Biol Biotechnol* 2017, **4**:13.
2. Seibold PS, Lenz C, Gressler M, Hoffmeister D: **The *Laetiporus* polyketide synthase LpaA produces a series of antifungal polyenes**. *J Antibiot (Tokyo)* 2020, **73**(10):711-720.
3. Lawrinowitz S, Wurlitzer JM, Weiss D, Arndt HD, Kothe E, Gressler M, Hoffmeister D: **Blue Light-Dependent Pre-mRNA Splicing Controls Pigment Biosynthesis in the Mushroom *Terana caerulea***. *Microbiol Spectr* 2022, **10**(5):e0106522.

Table S5. NMR spectroscopy data. ^{1}H and ^{13}C NMR spectroscopy data for phlebiopsin B (600 MHz for ^{1}H, 150 MHz for ^{13}C, CD_3OD, 300 K).

Position	δ_C	δ_H, mult. (J [Hz])	^{1}H-^{1}H COSY	HMBC ($^{1}H \rightarrow ^{13}C$)
1	199.6	–	–	6'
2	130.1	–	–	–
3	167.9	–	–	2'
4	199.1	–	–	2''
5	77.7	–	–	2'', 3'', 5'', 6''
1′	130.8	–	–	3', 5'
2′	130.3	8.14, d (7.6)	3'	4', 6'
3′	129.3	7.41, d (7.9)	2', 4'	–
4′	130.3	7.36, t (7.4)	3', 5'	2', 6'
5′	129.3	7.41, d (7.9)	4', 6'	–
6′	130.3	8.14, d (7.6)	5'	2', 4'
1′′	139.0	–	–	3'', 5''
2′′	127.0	7.42, m	3''	4'', 6''
3′′	129.6	7.32, t (7.5)	2'', 4''	5''
4′′	129.4	7.27, t (7.2)	3'', 5''	–
5′′	129.6	7.32, t (7.5)	4'', 6''	3''
6′′	127.0	7.42, m	5''	2'', 4''
5-OH	–	n.d.*	–	–

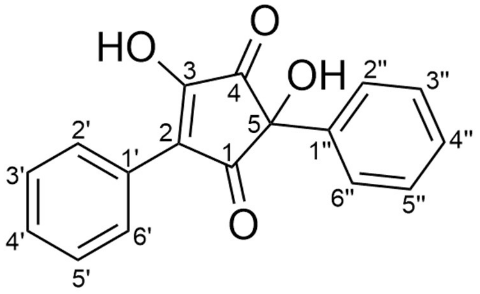

*Not detected

Table S6. Fungal strains and genotypes. JMRC = Jena Microbial Resource Collection; FGSC = Fungal Genetics Stock Center.

Strain	Genotype	Reference/ Source
Hapalopilus rutilans CBS 490.95	wild type	Westerdijk Institute
Psilocybe cubensis FSU12407	dikaryon	JMRC
Terana caerulea CBS 452.86	wild type	Westerdijk Institute
Serpula lacrymans S7	wild type	[1]
Aspergillus nidulans FGSC A4	wild type	FGSC
Aspergillus niger ATNT16Δ*pyrG*x24	TetOn:*terR_ble*; Δ*pyrG*::*ptrA*	[2]
Aspergillus niger tNAL000	TetOn:*terR_ble*; Δ*pyrG*::*ptrA*; P*terA*:*His*$_6$*_pyrG*	[3]
Aspergillus niger tPS10	TetOn:*terR_ble*; Δ*pyrG*::*ptrA*; P*terA*:*ppaA2_pyrG*	This study
Aspergillus niger tPS11	TetOn:*terR_ble*; Δ*pyrG*::*ptrA*; P*terA*:*ppaA1_pyrG*	This study
Aspergillus nidulans tPS15	P*alcA*:*His*$_6$*_ptrA*	This study
Aspergillus nidulans tPS18	P*alcA*:*ppaA2*:*His*$_6$*_ptrA*	This study
Aspergillus nidulans tPS19	P*alcA*:*ppaA1*:*His*$_6$*_ptrA*	This study
Aspergillus niger tPS20	TetOn:*terR_ble*; Δ*pyrG*::*ptrA*; P*terA*:*His*$_6$:*ppaA1*(S628A)*_pyrG*	This study
Aspergillus niger tPS21	TetOn:*terR_ble*; Δ*pyrG*::*ptrA*; P*terA*: *His*$_6$:*ppaA1*(D1188A)*_pyrG*	This study
Aspergillus niger tPS22	TetOn:*terR_ble*; Δ*pyrG*::*ptrA*; P*terA*: *His*$_6$:*ppaA1*(H1243A)*_pyrG*	This study
Aspergillus niger tPS23	TetOn:*terR_ble*; Δ*pyrG*::*ptrA*; P*terA*: *His*$_6$:*ppaA1*(H1186A_D1188A_H1243A)*_pyrG*	This study
Aspergillus niger tPS24	TetOn:*terR_ble*; Δ*pyrG*::*ptrA*; P*terA*: *His*$_6$:*ppaA1*ΔD*_pyrG*	This study
Aspergillus nidulans tPS28	P*alcA*:*ppaA1ΔD*:*His*$_6$*_ptrA*	This study
Aspergillus nidulans tPS29	P*alcA*:*ppaA1(A-T)*:*corA(TE)*:*His*$_6$*_ptrA*	This study
Aspergillus nidulans tPS30	P*alcA*:*corA(A-T)*:*ppaA1(TE)*:*His*$_6$*_ptrA*	This study
Aspergillus nidulans tPS31	P*alcA*:*corA(A-T-TE)*:*ppaA1(D)*:*His*$_6$*_ptrA*	This study
Aspergillus nidulans tPS32	P*alcA*:*corA(A-T)*:*ppaA1(TE-D)*:*His*$_6$*_ptrA*	This study
Aspergillus nidulans tPS35	P*alcA*:*corA*(I298N):*His*$_6$*_ptrA*	This study
Aspergillus nidulans tPS36	P*alcA*:*ppaA1*(V302N):*His*$_6$*_ptrA*	This study
Aspergillus nidulans tPS37	P*alcA*:*nps3*(N323I):*His*$_6$*_ptrA*	This study
Aspergillus nidulans tPS38	P*alcA*:*nps3*(N323V):*His*$_6$*_ptrA*	This study
Aspergillus nidulans tPS39	P*alcA*:*nps3*:*His*$_6$*_ptrA*	This study
Aspergillus nidulans tStL04	P*alcA*:*corA_ptrA*	[4]
Aspergillus nidulans tStL07	P*alcA*:*hapA1_ptrA*	This study
Aspergillus nidulans tStL08	P*alcA*:*hapA2_ptrA*	This study

References

1. Eastwood DC, Floudas D, Binder M, Majcherczyk A, Schneider P, Aerts A, Asiegbu FO, Baker SE, Barry K, Bendiksby M *et al*: **The plant cell wall-decomposing machinery underlies the functional diversity of forest fungi**. *Science* 2011, **333**(6043):762-765.

2. Geib E, Brock M: **ATNT: an enhanced system for expression of polycistronic secondary metabolite gene clusters in *Aspergillus niger***. *Fungal Biol Biotechnol* 2017, **4**:13.
3. Löhr NA, Eisen F, Thiele W, Platz L, Motter J, Hüttel W, Gressler M, Müller M, Hoffmeister D: **Unprecedented Mushroom Polyketide Synthases Produce the Universal Anthraquinone Precursor**. *Angew Chem Int Ed* 2022, **61**(24):e202116142.
4. Lawrinowitz S, Wurlitzer JM, Weiss D, Arndt HD, Kothe E, Gressler M, Hoffmeister D: **Blue Light-Dependent Pre-mRNA Splicing Controls Pigment Biosynthesis in the Mushroom *Terana caerulea***. *Microbiol Spectr* 2022, **10**(5):e0106522.

Table S7. Oligonucleotides used for expression analysis by qRT-PCR.

Oligonucleotide	5´-3´sequence	Target gene	Fragment size (gDNA) [bp]	Fragment size (cDNA) [bp]	Primer efficiency
oMG390	TCGGAATCGCTGAAAATGGC	*enoA*	186	120	1.00
oMG391	GGAGACGTTGGGAGCAAAGC				
oPS447	TGAAAGACCTGCTCACATCG	*ppaA2*	202	153	0.99
oPS448	TTCAGCTCCAAGTTCAGGATAC				
oPS453	TAATTCTCCTTCTCTGCGCG	*ppaA1*	178	125	1.03
oPS545	AGAAAGGCACATCTGGACTG				

Table S8. PCR methods.

Method	Reaction Mix		Thermal cycling		
	Component	Volume [µL]	Temperature [°C]	Time	Cycles
A	5 × PrimeSTAR GXL Buffer	10	98	10 s	
	2.5 mM dNTP Mix	4	60	15 s	35-40
	Primer forward 10 pmol µL^{-1}	1.5	68	1 min kb^{-1}	
	Primer reverse 10 pmol µL^{-1}	1.5	10	∞	1
	DNA template*	1			
	PrimeSTAR GXL DNA Polymerase (1.25 U µL^{-1})	1			
	dH_2O	to 50			
B	5 × Phusion GC Buffer	4	98	2 min	1
	10 mM dNTP Mix	0.4	98	30 s	
	Primer forward 10 pmol µL^{-1}	1	60	30 s	35-40
	Primer reverse 10 pmol µL^{-1}	1	72	30 s kb^{-1}	
	DNA template*	1	72	5-10 min	1
	Phusion DNA Polymerase (2 U µL^{-1})	0.1	10	∞	
	DMSO (optional)	1.5			
	dH_2O	to 20			
C	2 × Xtreme Buffer	25	94	2 min	1
	2 mM dNTP Mix	10	98	10 s	
	Primer forward 10 pmol µL^{-1}	1.5	60	30 s	35-40
	Primer reverse 10 pmol µL^{-1}	1.5	68	1 min kb^{-1}	
	DNA template*	1	68	5-10 min	1
	KOD Xtreme Hot Start DNA Polymerase (1 U µL^{-1})	1	10	∞	
	dH_2O	to 50	94		
D	10 × DreamTaq Buffer	2	95	2 min	1
	10 mM dNTP Mix	0.4	95	30 s	
	Primer forward 10 pmol µL^{-1}	0.4	60	30 s	31
	Primer reverse 10 pmol µL^{-1}	0.4	72	1 min kb^{-1}	
	DNA template*	X**	72	5-10 min	1
	DreamTaq Polymerase (5 U µL^{-1})	0.1	10	∞	
	dH_2O	to 20	95		
E	5 × Phusion HF Buffer	4	98	2 min	1
	10 mM dNTP Mix	0.4	98	30 s	
	Primer forward 10 pmol µL^{-1}	1	60	30 s	35-40
	Primer reverse 10 pmol µL^{-1}	1	72	30 s kb^{-1}	
	DNA template*	1	72	5-10 min	1
	Phusion DNA Polymerase (2 U µL^{-1})	0.2	10	∞	
	dH_2O	to 20			

*DNA template: 1 µL of 100 ng µL^{-1} gDNA, 10 ng µL^{-1} plasmid DNA, reverse transcription reaction (1:5 diluted with dH_2O), **or 10 µL water with resuspended *E. coli* cells (for colony PCR).

Table S9. Oligonucleotides used for cloning, colony PCRs, and DNA sequencing.

Oligonucleotide	5´-3´sequence	Target	Purpose
oMG116	GAGATGTGGTAGACGATTGATCC	pSMX2-URA (T*trpC* from *A. niger*)	sequencing/colony PCR
oMG169	GCGCTTACACAGTACACGAGG	pMG49 (T*trpC* from *A. oryzae*)	sequencing/colony PCR
oMG172	ATGGTCATGCGCCGTCCCGC	pPS29 (*ptrA*)	sequencing
oMG234	TTACGCCGGCGCGCCGTAGATATTTTCGAAGGGATTC	pMG49 (P*alcA*)	sequencing/colony PCR
oMG360	CCTCCAAGAGAGATCCAGAC	pSMX2-URA (P*terA*)	sequencing/colony PCR
oMG361	GAATTTTACCAGTGGCCTAGG	pSMX2-URA (T*trpC* from *A. niger*)	sequencing/colony PCR
oMG370	GATCCTCTCTCTGATATTGTCG	pSMX2-URA (P*terA*)	sequencing/colony PCR
oMG468	ACTTAACGTTACTGAAATCATCAAACAG	pMG49 (T*trpC* from *A. oryzae*)	vector amplicfication
oMG469	TTTGAGGCGAGGTGATAGGATTG	pMG49 (P*alcA*)	vector amplicfication
oMG474	CATCCCCGCATAGCTGAACATC	pPS29 (P*alcA*)	sequencing/ colony PCR
oPS355	TTGAAATCACTGCTGTTATCCATGTTAGACGATACCTAGCTGCCTTGC	*ppaA2*	cloning of pPS13
oPS356	CACCATGCATCATCATCACCATCACCATACCACTGAACCGACGACAA	*ppaA2*	cloning of pPS13
oPS357	CACCATGCATCATCATCACCATCACGAATCAACAGGGCCCACAAC	*ppaA1*	cloning of pPS14
oPS358	GAAATCACTGCTGTTATCCATGTTAAACAATTCCCAACTTCTTTGCC	*ppaA1*	cloning of pPS14
oPS362	ATCAAAACTCTAAGCGCCGA	*ppaA2*	colony PCR *ppaA2*
oPS363	ATAACCCTGAGCGCTGATAG	*ppaA1*	colony PCR *ppaA1*
oPS429	GGTGATGGTGATGATGTCGCGATCGGACGATACCTAGCTGCCTTG	*ppaA2*	cloning of pPS35
oPS430	TCCAATCCTATCACCTCGCCTCAAAATGCATACCACTGAACCGAC	*ppaA2*	cloning of pPS35
oPS431	TCCAATCCTATCACCTCGCCTCAAAATGGAATCAACAGGGCCCAC	*ppaA1*	cloning of pPS36 and pPS50
oPS432	GGTGATGGTGATGATGTCGCGATCGAACAATTCCCAACTTCTTTGCCTC	*ppaA1*	cloning of pPS36
oPS438	CTTAACTATGCGGCATCAGAGC	pMG49	cloning of pPS29
oPS439	GCTCTGATGCCGCATAGTTAAG	pMG49	cloning of pPS29
oPS441	TCGCGACATCATCACCATCACCATTAGACTTAACGTTACTGAAATCATCAAACAG	pMG49	cloning of pPS29
oPS442	AATGGTGATGGTGATGATGTCGCGATCGCATTTTGAGGCGAGGTGATAGGA	pMG49	cloning of pPS29
oPS459	ACCAGGATAGAGAGCATTGTCTG	pSMX2-URA (P*pyrG*)	sequencing
oPS460	CAGACAATGCTCTCTATCCTGGT	pSMX2-URA (P*pyrG*)	sequencing

Table S9 (continued). Oligonucleotides used for cloning, colony PCRs, and DNA sequencing.

Oligonucleotide	5´-3´sequence	Target	Purpose
oPS461	GCTTCTGCCATGCATCTCATAC	*ppaA1*	cloning of pPS37 (*ppaA1*_S628A)
oPS462	GTATGAGATGCATGGCAGAAGC	*ppaA1*	cloning of pPS37 (*ppaA1*_S628A)
oPS465	CATCGCGCTCCTTCCACATT	*ppaA1*	cloning of pPS39 (*ppaA1*_D1188A)
oPS466	AATGTGGAAGGAGCGCGATG	*ppaA1*	cloning of pPS39 (*ppaA1*_D1188A)
oPS467	CCACCACTGCTCGAGTAAATAC	*ppaA1*	cloning of pPS40 (*ppaA1*_H1243A)
oPS468	GTATTTACTCGAGCAGTGGTGG	*ppaA1*	cloning of pPS40 (*ppaA1*_H1243A)
oPS469	AATGCACGAGGCTCGCGCTC	*ppaA1*	cloning of pPS41 (*ppaA1*_H1186A_D1188A_H1243A)
oPS470	GAGCGCGAGCCTCGTGCATT	*ppaA1*	cloning of pPS41 (*ppaA1*_H1186A_D1188A_H1243A)
oPS471	ATTGAAATCACTGCTGTTATCCATGTTAAGAGTCAAAATTCTGTCGGGAA	*ppaA1*	cloning of pPS42 (*ppaA1* D domain deletion)
oPS472	ATCACACTAGCCAAGGCGTA	*ppaA1*	sequencing
oPS473	AAGATGAGAACTTCGCCGAC	*ppaA1*	sequencing
oPS474	CAAATCAATGGCCCAAGGAC	*ppaA1*	sequencing
oPS490	GGTGATGGTGATGATGTCGCGATCGAGAGTCAAAATTCTGTCGGGAA	*ppaA1*	cloning of pPS50 (*ppaA1* D-domain deletion)
oPS491	CGAGGAAGACAGGCGGCTTCGATCCCTGAGGGTTCAGGCAAAGAAG	*ppaA1*	cloning of pPS51 (*ppaA1*/*corA* domain swap)
oPS492	GGATCGAAGCCGCCTGTCTT	*corA*	cloning of pPS51 (*ppaA1*/*corA* domain swap)
oPS493	GGTGATGGTGATGATGTCGCGATCGCAGCGCAGCACTCGCGC	*corA*	cloning of pPS51 (*ppaA1*/*corA* domain swap)
oPS494	TCCAATCCTATCACCTCGCCTCAAAATGCTATATAATCTCTCCTCGCCCG	*corA*	cloning of pPS52, pPS53 and pPS54 (*ppaA1*/*corA* domain swap)
oPS495	CAAGGTAAATGGGTGGCTTTGAGCCGTGCGGGTTCATGCAGACCA	*corA*	cloning of pPS52 and pPS54 (*ppaA1*/*corA* domain swap)
oPS496	CCCGCTGGTCTGCATGAACCCGCACGGCTCAAAGCCACCCATTTA	*ppaA1*	cloning of pPS52 and pPS54 (*ppaA1*/*corA* domain swap)
oPS497	GGTGATGGTGATGATGTCGCGATCGAGAGTCAAAATTCTGTCGGGAA	*ppaA1*	cloning of pPS52 (*ppaA1*/*corA* domain swap)
oPS498	CGTCCCTCATCTTAGCGGCGAAGGACACGACGTGCTCCTCGGAGA	*corA*	cloning of pPS53 (*ppaA1*/*corA* domain swap)
oPS499	GATGCTCTCCGAGGAGCACGTCGTGTCCTTCGCCGCTAAGATGAG	*ppaA1*	cloning of pPS53 (*ppaA1*/*corA* domain swap)
oPS500	GGTGATGGTGATGATGTCGCGATCGAACAATTCCCAACTTCTTTGCCT	*ppaA1*	cloning of pPS53 and pPS54 (*ppaA1*/*corA* domain swap)
oPS501	CTTCCATGCATCTCATACGTCTC	*ppaA1*	sequencing
oPS502	CGTCTGCAGGTGCTTGAAGA	*corA*	colony PCR
oPS503	ATCACGAAGCTCACGCGGAT	*corA*	sequencing
oPS504	TGGCTCGAGGTGCTCATCAA	*corA*	sequencing

Table S9 (continued). Oligonucleotides used for cloning, colony PCRs, and DNA sequencing.

Oligonucleotide	5´-3´sequence	Target	Purpose
oPS574	GCTTGGACGCGTTCAACTC	*corA*	cloning of pPS57 (*corA*_I298N)
oPS575	GAGTTGAACGCGTCCAAGC	*corA*	cloning of pPS57 (*corA*_I298N)
oPS576	AATCCTATCACCTCGCCTCAAAATGGCCCCAGCCCCGACATC	*nps3*	cloning of pPS59, pPS60 and pPS61
oPS577	GGTGATGGTGATGATGTCGCGATCGAACTCCACGGGCTTCAAGAC	*nps3*	cloning of pPS59, pPS60 and pPS61
oPS578	TGAAACGCATCATCAGTGGTG	*nps3*	cloning of pPS59 (*nps3*_N323I)
oPS579	CACCACTGATGATGCGTTTCA	*nps3*	cloning of pPS59 (*nps3*_N323I)
oPS580	TGAAACGCATCGTCAGTGGTG	*nps3*	cloning of pPS60 (*nps3*_N323V)
oPS581	CACCACTGACGATGCGTTTCA	*nps3*	cloning of pPS60 (*nps3*_N323V)
oPS582	GTTATGAACACTGGAGGAGAGG	*ppaA1*	cloning of pPS58 (*ppaA1*_V302N)
oPS583	CCTCTCCTCCAGTGTTCATAAC	*ppaA1*	cloning of pPS58 (*ppaA1*_V302N)
oPS584	TAACGGAAGCCAATACCACG	*corA*	sequencing
oPS585	TCAAATCCGCAAAGTCCACG	*corA*	sequencing, colony PCR
oPS586	CTCGAGATGCACATAACACCTT	*nps3*	sequencing
oPS587	GACCACAAAAGAGACCTTCGAG	*nps3*	sequencing, colony PCR
oPS588	CACGTCACTCACACTTTCAGTC	*ppaA1*	sequencing
oPS589	CAGTTTCAGTCATCCCAAACG	*ppaA1*	sequencing, colony PCR
oSS189	CAATCCTATCACCTCGCCTCAAAATGGCCGCAACATCCTCTACACTTCC	*hapA1*	cloning of pSS10
oSS190	GCTGTTTGATGATTTCAGTAACGTTAAGTTTACGAGCTGGTAGTAGGG	*hapA1*	cloning of pSS10
oSS191	CAATCCTATCACCTCGCCTCAAAATGAGCACCTCATTCAACCCCTCC	*hapA2*	cloning of pSS11
oSS192	GCTGTTTGATGATTTCAGTAACGTTAAGTCTACAGCTGCTTGGCTGCGCG	*hapA2*	cloning of pSS11

Table S10. HPLC and flash chromatography methods.

Method	Column	Parameters	Eluents	Gradient	
				Time [min]	B [%]
A	Macherey-Nagel<br>EC UHPLC column,<br>Nucleodur C18 Gravity,<br>50 × 2 mm, 1.8 µm	T: 30°C<br>flow: 1 mL min^{-1}	A: water + 0,1% formic acid<br>B: acetonitrile	0.0<br>4.0<br>4.5<br>5.0	5<br>72<br>95<br>95
B	Büchi<br>FlashPure Silica 40 g<br>40 µm irregular	flow: 45 mL min^{-1}	A: dichloromethane<br>B: methanol	0.0<br>8.0<br>16.0<br>20.0<br>32.0	0<br>0<br>20<br>100<br>100
C	Büchi<br>FlashPure ID C18 12 g<br>40 µm irregular	flow: 30 mL min^{-1}	A: water + 0,1% formic acid<br>B: acetonitrile	0.0<br>2.0<br>5.0<br>8.0<br>10.0<br>12.0<br>16.0	0<br>0-20<br>20-40<br>40-60<br>60-80<br>80-100<br>100
D	Agilent<br>ZORBAX Eclipse XDB C18<br>250 × 9.4 mm, 5 µm	T: 12°C<br>flow: 2 mL min^{-1}	A: water + 0,1% formic acid<br>B: acetonitrile	0.0<br>9.0<br>9.5<br>20.0<br>20.5<br>25.0<br>26.5<br>28.5	30<br>30<br>35<br>35<br>40<br>40<br>100<br>100
E	Thermo Scientific<br>Accucore C18<br>100 × 2.1 mm, 2.6 µm	flow: 0.2 mL min^{-1}	A: water + 0,1% formic acid<br>B: acetonitrile	0.0<br>7.0	5<br>98
F	Thermo Scientific<br>Accucore C18<br>100 × 2.1 mm, 2.6 µm	flow: 0.2 mL min^{-1}	A: water + 0,1% formic acid<br>B: acetonitrile	0.0<br>10.0	5<br>98
