# Supplementary material for: Bifurcate evolution of quinone synthetases in basidiomycetes
Source: Fungal Biol Biotechnol. 2023 Jul 3;10:14. doi: 10.1186/s40694-023-00162-1 (PMC10316625; doi:10.1186/s40694-023-00162-1)
Supplement: Supplementary file 2 — Additional file 2. 23 supplementary figures with supporting biological and chemical data. [file 40694_2023_162_MOESM2_ESM.pdf]

Organism	Enzyme	NRPS code					Substrate	Accession#
		1 2 3	4	5 6	7 8 9	10		
P. cubensis	PpaA1	^{212}LNWINFDHVANVTEAHLHAI^{231}	^{261}VTHTFSPNFLLAQI^{274}	^{298}LRVMVTGGEA^{307}	^{330}LRAAFGMTETGAGIIYN^{346}	^{549}TALGKVSR^{556}	PP	OQ821699
T. caerulea	CorA	^{213}LNWIAFDHVACVSEVHLHAL^{232}	^{262}ITYTFSPNFLVAQI^{275}	^{294}LDAFISGGEA^{303}	^{326}LRAGFGMSETGAGSIYD^{342}	^{540}TALDKVSR^{547}	PP	UVV38562.1
H. rutilans	HapA1	^{206}LNWIAFDHVACVSEIHLHAL^{225}	^{255}IAYTFSPNFLMAHI^{268}	^{290}MIAFISGGEA^{299}	^{322}LRAGFGMSETGAGCIYD^{338}	^{535}TALGKVSR^{542}	PP	OQ784619
H. rutilans	HapA2	^{212}LNWIAFDHVASVSEVHIQAL^{231}	^{263}ITYTFSPNFLIAQI^{276}	^{295}VQVFVSGGEA^{304}	^{327}LRAGFGMSETGAGCIYD^{343}	^{522}SALGKVSR^{529}	PP	OQ784620
T. panuoides	AtrA	^{233}LNWVGFDHVAGSLEMHIAPL^{252}	^{282}IDIAFAPNFLLAKM^{295}	^{314}LRRMNSGGEA^{323}	^{350}VAAGFGMTETCAGCIYD^{364}	^{556}TTLGKLSR^{563}	4-HPP	ACH90386.1
S. lacrymans	NPS3	^{238}LNWVGFDHVAGSLEMHITPL^{257}	^{287}INIAFAPNFLLAKL^{300}	^{319}VKRINSGGEA^{328}	^{355}VSAGFGMTETCAGCIYD^{371}	^{561}TTLGKLSR^{568}	4-HPP	EGO23141.1
P. involutus	InvA1	^{235}LNWVGFDHVAGSLEMHIAPL^{254}	^{284}INVAFAPNFLLAKL^{297}	^{316}VTRINSGGEA^{325}	^{352}ISPGFGMTETCAGCIYN^{368}	^{558}TTLGKLSR^{565}	4-HPP	A0A0S2E7Z1
P. involutus	InvA2	^{230}MNWVGFDHVAGSLEMHIAPL^{249}	^{279}IQLAFAPNFLLAKL^{292}	^{311}IKRINSGGEA^{320}	^{346}ISAGFGMTETCAGCIYD^{362}	^{552}TTLGKLSR^{559}	4-HPP	A0A0S1RUN4
P. involutus	InvA5	^{230}MNWVGFDHVAGSLEMHIAPL^{249}	^{279}IQLAFAPNFLLAKL^{292}	^{311}IKRINSGGEA^{320}	^{346}ISAGFGMTETCAGCIYD^{362}	^{552}TTLGKLSR^{559}	4-HPP	A0A0S2E7W7
S. grevillei	GreA	^{234}LNWVGFDHVAGSLEMHITPL^{253}	^{283}IELAFAPNFLLSKL^{296}	^{315}IKRINSGGEA^{324}	^{351}ISAGFGMTETCAGCIYD^{367}	^{557}TTLGKLSR^{564}	4-HPP	AFB76152
A. nidulans	MicA	^{218}YNWVGLDHVASLTEIHLHAL^{237}	^{267}VAYTFAPNFFLTKV^{280}	^{297}LKALISGGES^{306}	^{329}IRPGFGMTETCAGSIYS^{345}	^{536}SSLGKISH^{543}	PP	Q5B7T4
A. sarcoides	AcyN	^{207}LNWIGLDHVANLTETHLQAM^{226}	^{256}ITVTFAPNFFIASV^{269}	^{290}FRIMISGGEA^{299}	^{322}IRPGFGMTETCAGSIYS^{338}	^{527}SSLGKLSR^{535}	PP	P9WES4.1
A. terreus	PgnA	^{220}LNWVGMNHVASLVEVHLFSI^{239}	^{269}VSRTFAPNFFLAKL^{282}	^{302}LRYIVSGGEA^{311}	^{334}IVPAFGMTETCAGAIYN^{350}	^{540}STLGKLAR^{547}	PP	Q0CBN5
A. terreus	AtrA	^{200}MNWIRLDHVAALTEIHLPAI^{219}	^{249}VAKTFAPNFFLATL^{262}	^{281}L-YIFSGGEG^{289}	^{312}IVPGFGMTETCAGAINN^{328}	^{518}SALGKLSR^{525}	4-HPP	Q0CT94
A. terreus	MelA	^{205}LNWVGLDHVAAIIEIHLQAL^{224}	^{254}VSRTFAPNFFLARI^{267}	^{288}LRYVASGGEA^{297}	^{320}IVPGFGMTETCAGAIFN^{336}	^{524}STLGKLSR^{531}	4-HPP	A0A336U965
A. terreus	ApvA	^{207}LNWVGLDHVAAIVEIHLQAM^{226}	^{256}VSRTFAPNFFLAKL^{269}	^{290}LEYVASGGEA^{299}	^{322}IVPGFGMTETCAGSIFN^{338}	^{524}SSLGKLSR^{531}	4-HPP	Q0CWD0
A. terreus	BtyA	^{209}LNWVAFDHIASLAEMHFHPI^{228}	^{258}VGITFAPNFLLAKL^{271}	^{291}LMHLLSGGEA^{300}	^{324}IKPAFGMTETCAGCSFN^{340}	^{530}SSLGKISR^{537}	4-HPP	Q0CU19
A. terreus	AtqA	^{233}LSWVHMDHVANLVHCHLFAI^{252}	^{282}VSRTFAPNFLFAKL^{295}	^{315}ALYLDTGGEA^{324}	^{347}FKPSFGMTETCAGCIFN^{363}	^{552}TTLGKLSR^{559}	IP	Q0D034
A. nidulans	TdiA	^{231}LSWVHMDHVANLVHCHIFAI^{250}	^{280}VSRTFMPNFLCAKL^{293}	^{314}TLYIDTGGEA^{323}	^{346}FKPSFGMTETVAGCIFN^{362}	^{552}STLGKLSR^{559}	IP	CBF80711.1
Streptomyces sp.	EchA	^{241}FNWISYDHVAALLEAHMLPL^{260}	^{290}VTMTFTPNFLLGPL^{303}	^{326}LRHIVSGGEA^{335}	^{358}LWPAFGMTETCAGSIYN^{374}	^{558}TSLGKTLR^{565}	PP	AHN91924
R. solanacearum	RalA	^{219}LNWISFDHVAALLEAHLLPL^{238}	^{268}VSMTFAPNFLFGQI^{281}	^{303}LRHIISGGEA^{312}	^{335}LWPAFGMSETCAGSIYS^{351}	^{535}TSLGKIQR^{542}	PP	AEC03968.1
B. brevis	GrsA	^{227}GQFASISFDASVWEMFMALL^{246}	^{274}ITVITLPPTYVVHL^{287}	^{295}IQTLITAGSA^{304}	^{321}NAYGPTETTICATTWVA^{337}	^{513}TSNGKIDR^{520}	Phe	P0C062.1
		235 236 239	278	299 301	322 330 331	517		

Figure S1. Adenylation domain specificity code. Amino acid sequences of the A domains of PpaA1, CorA, HapA1, HapA2, and related enzymes were aligned with ClustalW [1]. Specificity code positions 1-10 are numbered according to the GrsA sequence [2]. The amino acid on position 5 and the substrate of the respective enzyme are highlighted in matching colors. Green: Val/Ile – phenylpyruvic acid (PP); cyan: Asn in basidiomycete enzymes, Phe/Ala/Leu in ascomycete enzymes – 4-hydroxyphenylpyruvic acid (4-HPP); blue: Asp – indole-3-pyruvic acid (IP); grey: Ile – phenylalanine (Phe).

References

1. Larkin MA, Blackshields G, Brown NP, Chenna R, McGettigan PA, McWilliam H, Valentin F, Wallace IM, Wilm A, Lopez R *et al*: **Clustal W and Clustal X version 2.0**. *Bioinformatics* 2007, **23**(21):2947-2948.
2. Conti E, Stachelhaus T, Marahiel MA, Brick P: **Structural basis for the activation of phenylalanine in the non-ribosomal biosynthesis of gramicidin S**. *EMBO J* 1997, **16**(14):4174-4183.

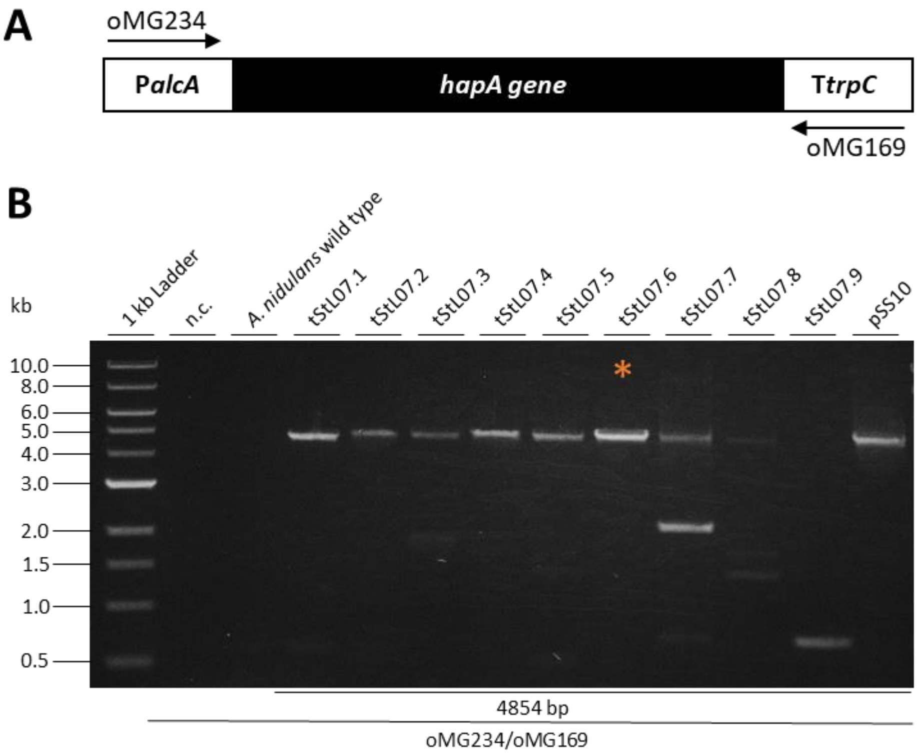

Figure S2. PCR to verify *H. rutilans* transgene integration in the *A. nidulans* genome. (**A**) Schematic representation of the oligonucleotide binding sites of oMG234 in P*alcA* and oMG169 in T*trpC.* (**B**) Full integration of *hapA1* in the *A. nidulans* genome was verified by PCR using oligonucleotide pairs oMG234/oMG169. DNA of plasmid pSS10 (encoding *hapA1*) was used as positive control. (**C**) Full integration of *hapA2* in the *A. nidulans* genome was verified by PCR using oligonucleotide pairs oMG234/oMG169. DNA of plasmid pSS11 (encoding *hapA2*) was used as positive control. Negative controls were dH_2O (n.c.) and gDNA of the *A. nidulans* FGSC A4 (wild type) parental strain. PCR amplicons were visualized in ethidium bromide-stained agarose gels. Orange asterisks indicate the transformants chosen for further work.

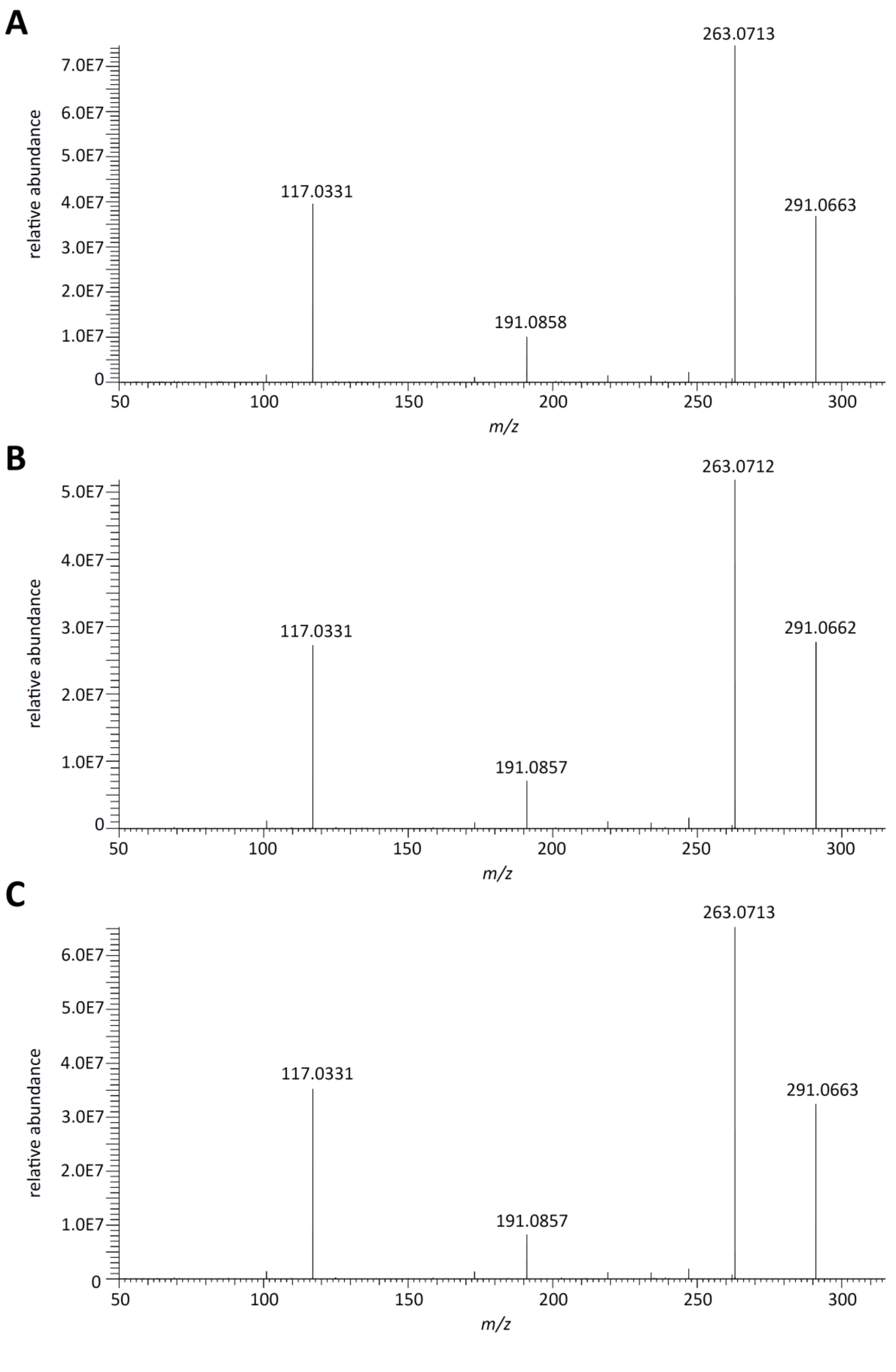

Figure S3. MS/MS fragmentation of polyporic acid, produced by (**A**) *H. rutilans*, (**B**) *A. nidulans* tStL07 and (**C**) *A. nidulans* tStL08. The spectra were recorded in negative ionization mode.

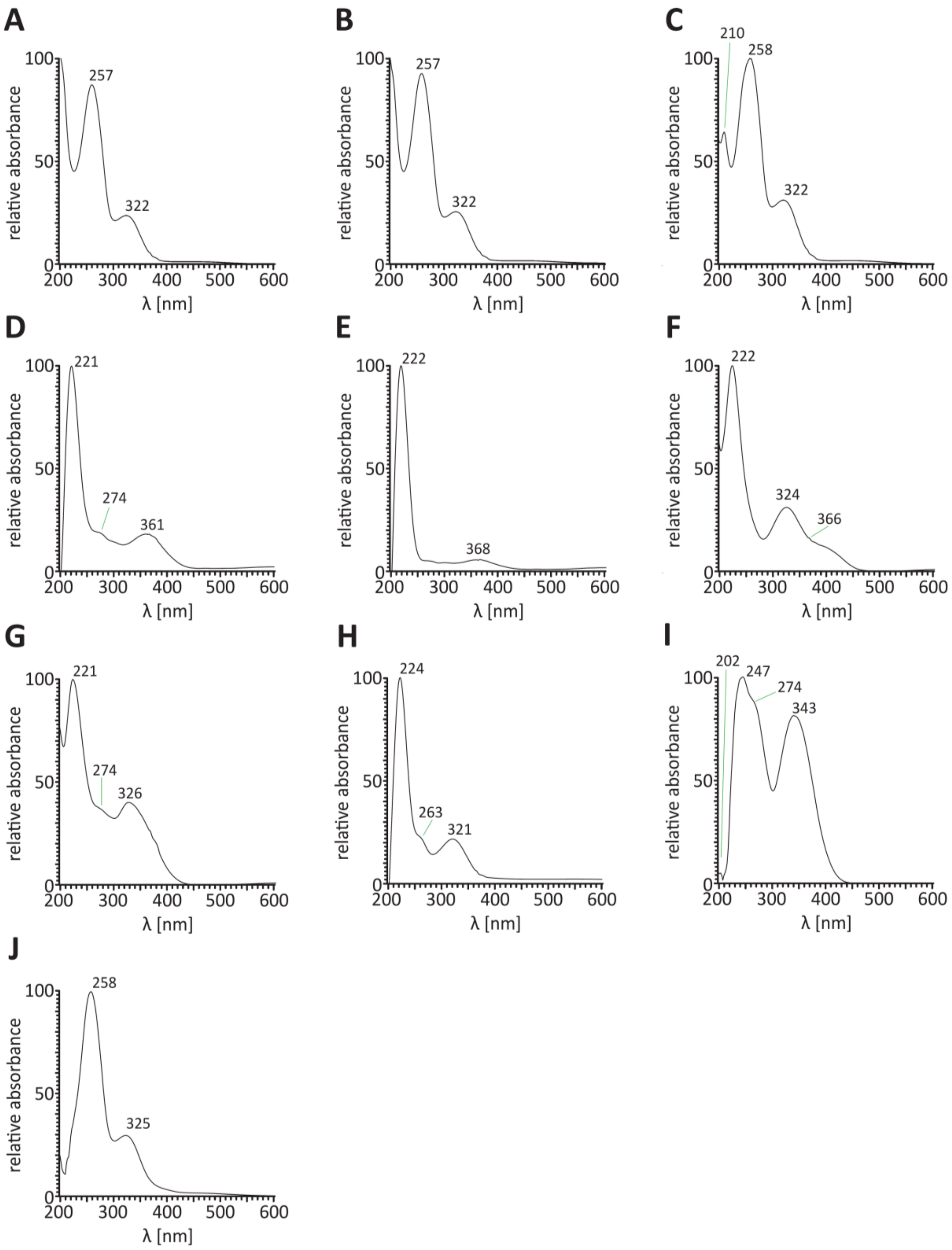

Figure S4. UV/Vis spectra. Dried extracts were dissolved in methanol. (**A**) Polyporic acid detected in an *H. rutilans* ethyl acetate extract; (**B**) polyporic acid in an ethyl acetate extract of *A. nidulans* tStL07 and (**C**) *A. nidulans* tStL08; (**D-H**) UV/Vis spectra (according to the five peaks *, **, (1), (2) and (3) from Figure 5) of an ethyl acetate extract of *A. niger* tPS11; (**I**) purified phlebiopsin B from *A. niger* tPS11; (**J**) synthetic polyporic acid standard.

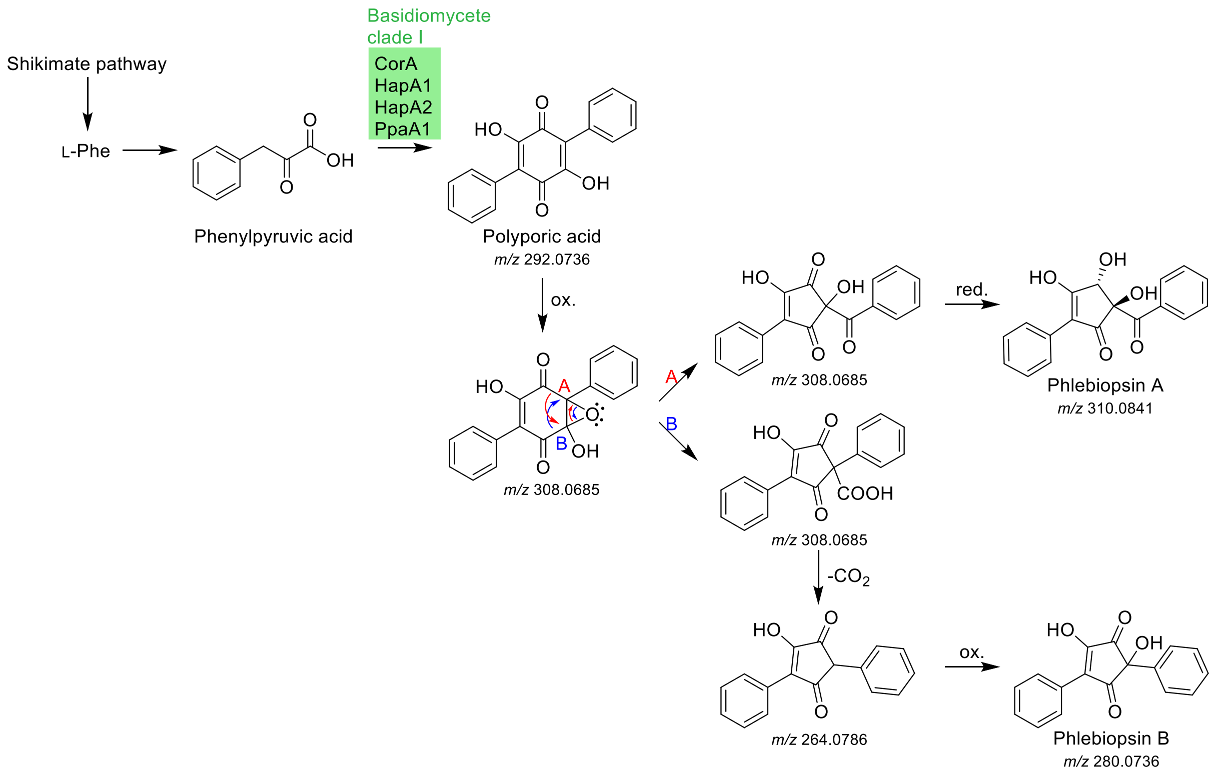

Figure S5. Biosynthetic route to phlebiopsins as proposed by Kälvö et al. [1] After epoxidation of polyporic acid, the pathway hypothetically diverges by two alternative ring opening reactions (A and B). Corresponding masses were detected by mass spectrometry in negative mode: polyporic acid *m/z* 291.0663 [M-H]$^-$; intermediates *m/z* 307.0612 and 263.0714 [M-H]$^-$; phlebiopsin A *m/z* 309.0768 [M-H]$^-$; phlebiopsin B *m/z* 279.0663 [M-H]$^-$.

References

1. Kälvö D, Menkis A, Broberg A: **Secondary Metabolites from the Root Rot Biocontrol Fungus *Phlebiopsis gigantea***. *Molecules* 2018, **23**(6).

Organism	Enzyme	Sequence		PDB ID
P. cubensis	PpaA1	^{1151}FPSPHEFNA----------ALWHYLPLTPEMKASEREGFVNGMHEHRDPSTFLTCLIQ-SRPGLQV^{1205}	^{1232}RLTGGKLVATTHRVNTLKIDND----RYTIPYVLTTK^{1264}	-
T. stipitatus	TropC	^{171}FGHPSDVLNDILINPSIPMRLLHYAPQENP----DPRQF--GVGDHTDFGCVSILLQQKGTKGLEV^{230}	^{258}RWTGGYYRSARHRVYITGER------RYSVAFFLNGN^{288}	6XJJ
N. crassa	T7H	^{178}FDSFVDVGDNIL-------RLLHYPAVKSEVFKINPGQV--RAGEHTDYGSITLLFQD-SRGGLQV^{233}	^{260}RWSNDTIKSTVHRVVEPPKQEDVHPPRYSIAYFCNPN^{296}	5C3Q

Figure S6. Alignment of amino acid sequences of fungal dioxygenases. Shown are portions of the dioxygenase domain of *Psilocybe cubensis* PpaA1 and the fungal dioxygenases TropC [1] and T7H [2]. Color codes of conserved residues: green: interaction with α-keto glutaric acid; red: Fe^{2+} binding $HxD(x)_nH$ motif; blue: acceptor substrate binding.

References

1. Doyon TJ, Skinner K, Yang D, Mallik L, Wymore T, Koutmos M, Zimmerman PM, Narayan A: **Radical tropolone biosynthesis**. *ChemRxiv* 2020.
2. Li W, Zhang T, Ding J: **Molecular basis for the substrate specificity and catalytic mechanism of thymine-7-hydroxylase in fungi**. *Nucleic Acids Res* 2015, **43**(20):10026-10038.

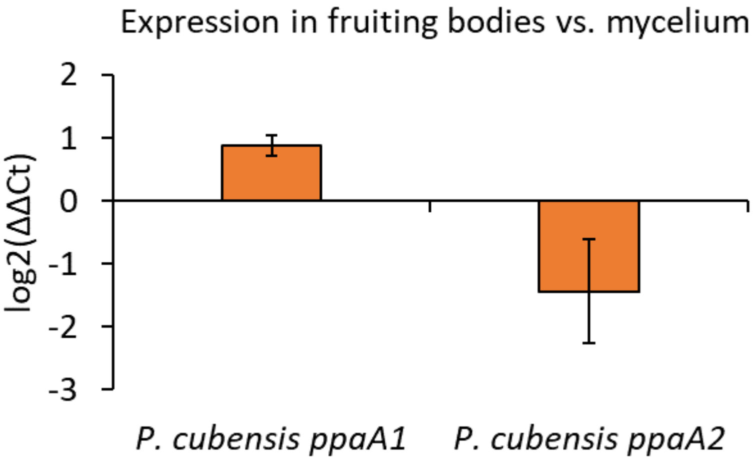

Figure S7. qRT-PCR of *P. cubensis ppaA1* and *ppaA2* gene expression. The gene expression in fruiting bodies, compared to mycelium, was analyzed. Shown values represent log2 fold changes and standard errors of means. The values are normalized to the expression of *enoA* as a control gene.

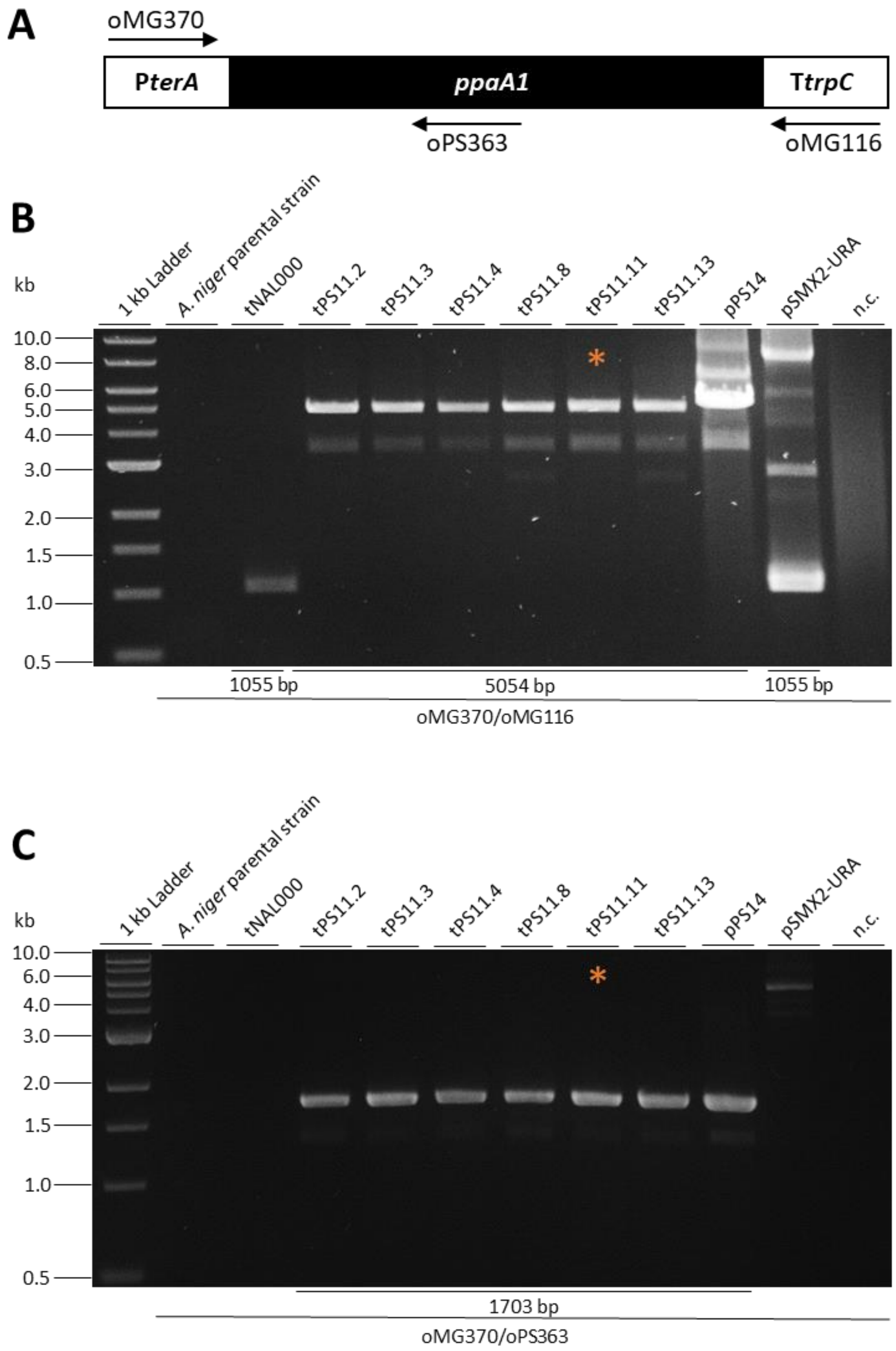

Figure S8. PCR to verify *P. cubensis ppaA1* integration into the *A. niger* genome. (**A**) Schematic representation of the oligonucleotide binding sites of oMG370 in P*alcA*, oPS363 in *ppaA1* and oMG116 in T*trpC.* (**B**) Full length **i**ntegration of *ppaA1* in the *A. niger* genome was verified by PCR using oligonucleotide pairs oMG370/oMG116 (targeting P*terA* and T*trpC*). (**C**) Moreover, oMG370/oPS363 (targeting P*terA* and *ppaA1*) were used to verify the integration of *ppaA1*. dH_2O served as negative control (n.c.). gDNA of the *A. niger* ATNTΔ*pyrG*24x parental strain, tNAL000 empty vector control and the empty vector pSMX2-URA were used as additional negative controls. Plasmid pPS14 (encoding *ppaA1*) was used as positive control. PCR amplicons were visualized in ethidium bromide-stained agarose gels. Orange asterisks indicate the transformants chosen for further work.

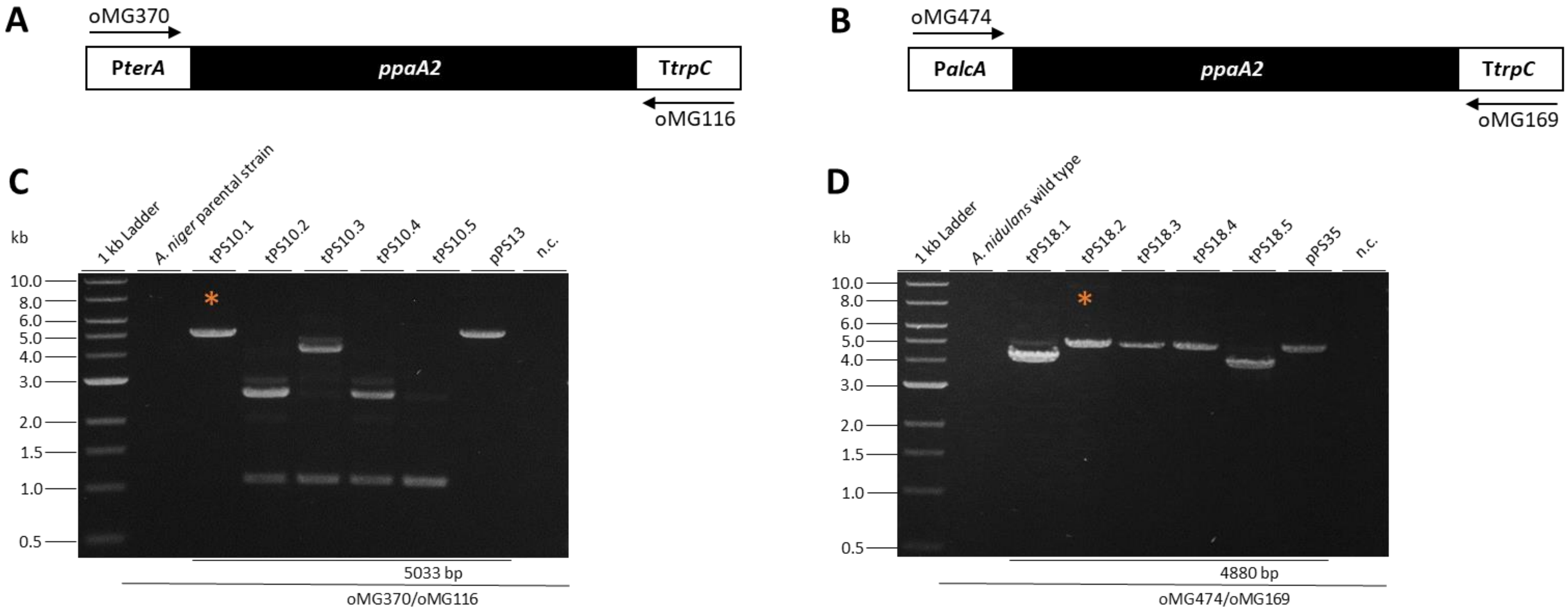

Figure S9. PCR to verify *P. cubensis ppaA2* integration into the *A. niger* and *A. nidulans* genomes. (**A**) Integration of *ppaA2* in the *A. niger* genome was verified by PCR using oligonucleotide pair oMG370/oMG116 (which target P*terA* and T*trpC*). (**B**) Integration of *ppaA2* in the *A. nidulans* host genome was verified by PCR using oligonucleotides oMG474 and oMG169 (which target P*alcA* and T*trpC*). dH$_2$O (n.c., negative control), gDNA of the *A. niger* ATNTΔ*pyrG*24x parental strain (in **C**) and gDNA of the *A. nidulans* FGSC A4 parental strain (wild type, in **D**) served as negative controls. Plasmids pPS13 and pPS35 (both encoding *ppaA2*) were used as positive controls. PCR amplicons were visualized in ethidium bromide-stained agarose gels. Orange asterisks indicate the transformants chosen for further work.

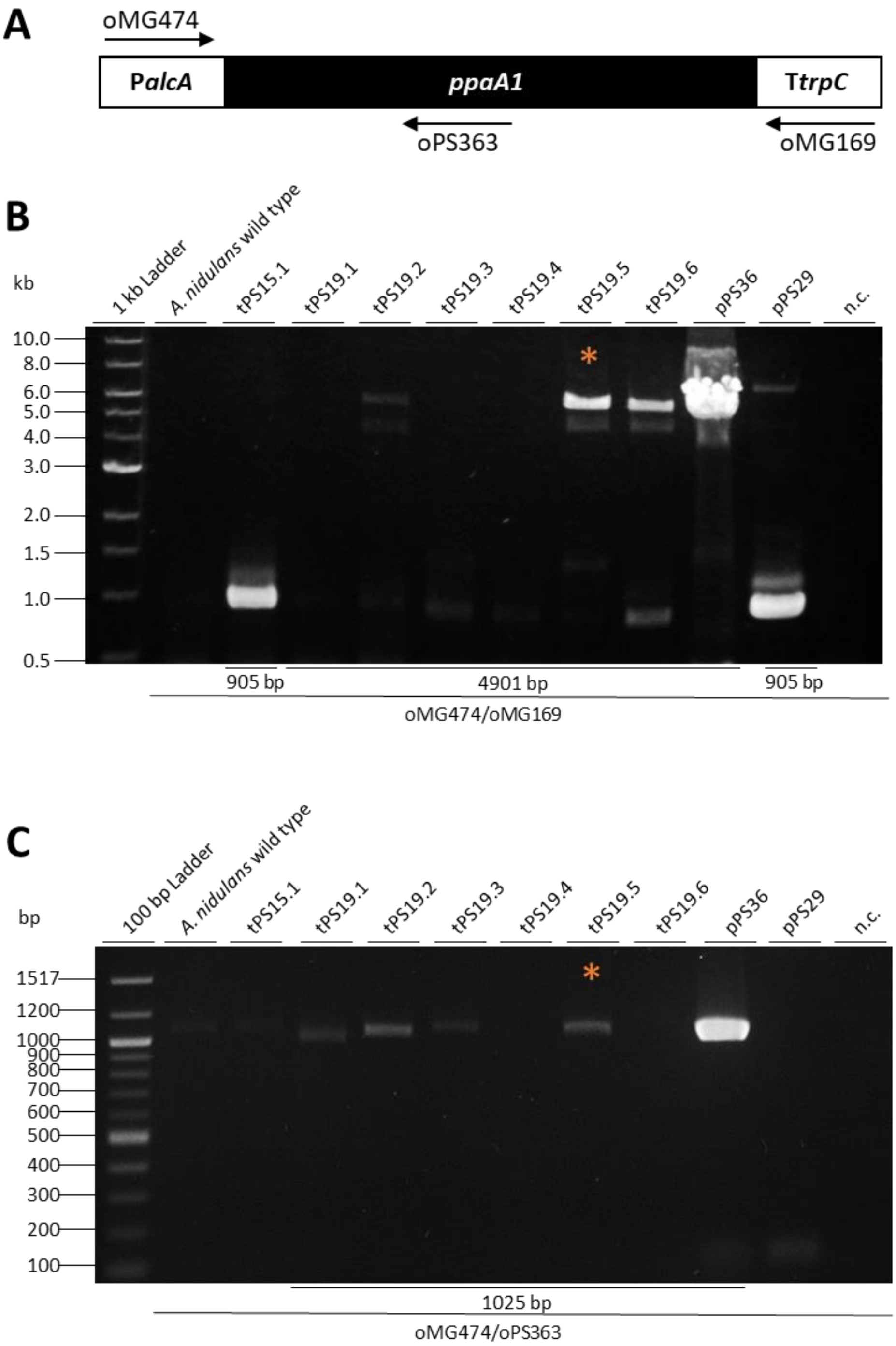

Figure S10. PCR to verify *P. cubensis ppaA1* integration into the *A. nidulans* genome. (**A**) Schematic representation of the oligonucleotide binding sites of oMG474 in P*alcA*, oPS363 in *ppaA1* and oMG169 in T*trpC.* (**B**) Full length integration of *ppaA1* in the *A. nidulans* host genome was verified by PCR using oligonucleotide pairs oMG474/oMG169 (targeting P*alcA* and T*trpC*). (**C**) Moreover, oMG474/oPS363 (targeting P*alcA* and *ppaA1*) were used to verify the *ppaA1* integration. dH_2O served as negative control (n.c.). gDNA of the *A. nidulans* FGSC A4 parental strain (wild type), tPS15 empty vector control and DNA of pPS29 empty vector were used as negative controls. DNA of plasmid pPS36 (encoding *ppaA1*) was used as positive control. PCR amplicons were visualized in ethidium bromide-stained agarose gels. Orange asterisks indicate the transformants chosen for further work.

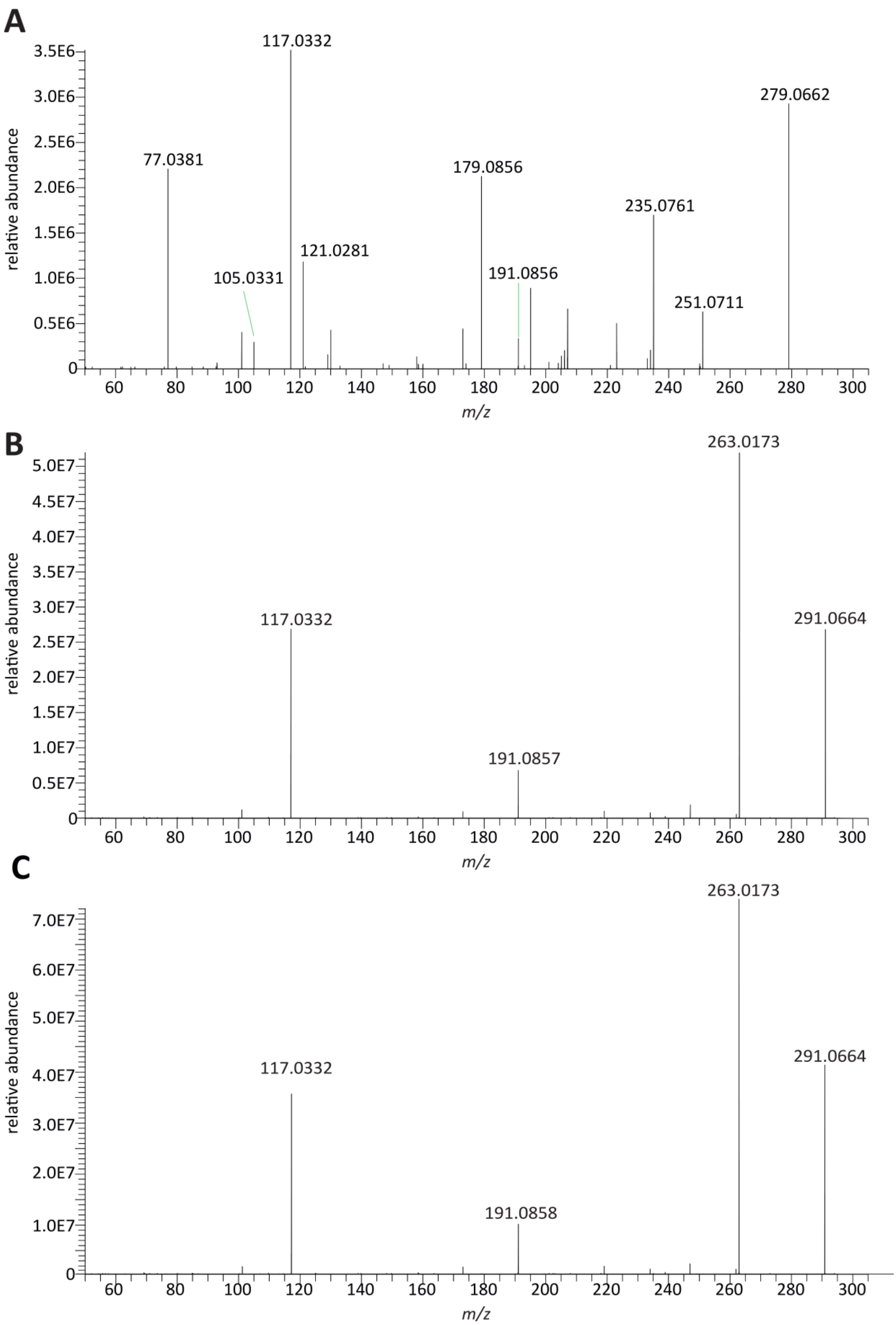

Figure S11. MS/MS fragmentation of major compounds of *A. nidulans* tPS19 and of synthetic polyporic acid. (A) phlebiopsin B and (**B**) polyporic acid produced by tPS19; (**C**) polyporic acid standard. The spectra were recorded in negative ionization mode.

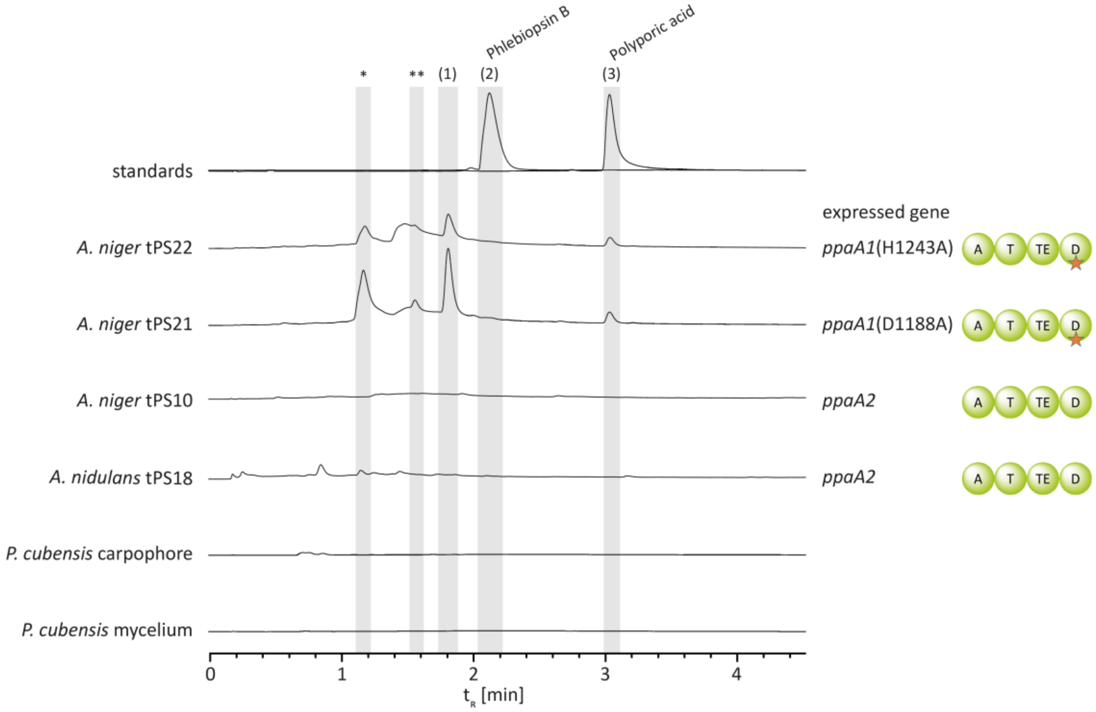

Figure S12. Additional chromatograms. Product formation by wild type and mutated *Psilocybe cubensis* quinone synthetases. Chromatograms were extracted at λ = 350 nm. Chromatograms of synthetic polyporic acid and of authentic phlebiopsin B, of ethyl acetate extracts of *P. cubensis* vegetative mycelium and fruiting bodies, and of ethyl acetate extracts of the culture broth of *Aspergillus niger* and *A. nidulans* expressing *ppaA2* or mutated *ppaA1* variants are shown.

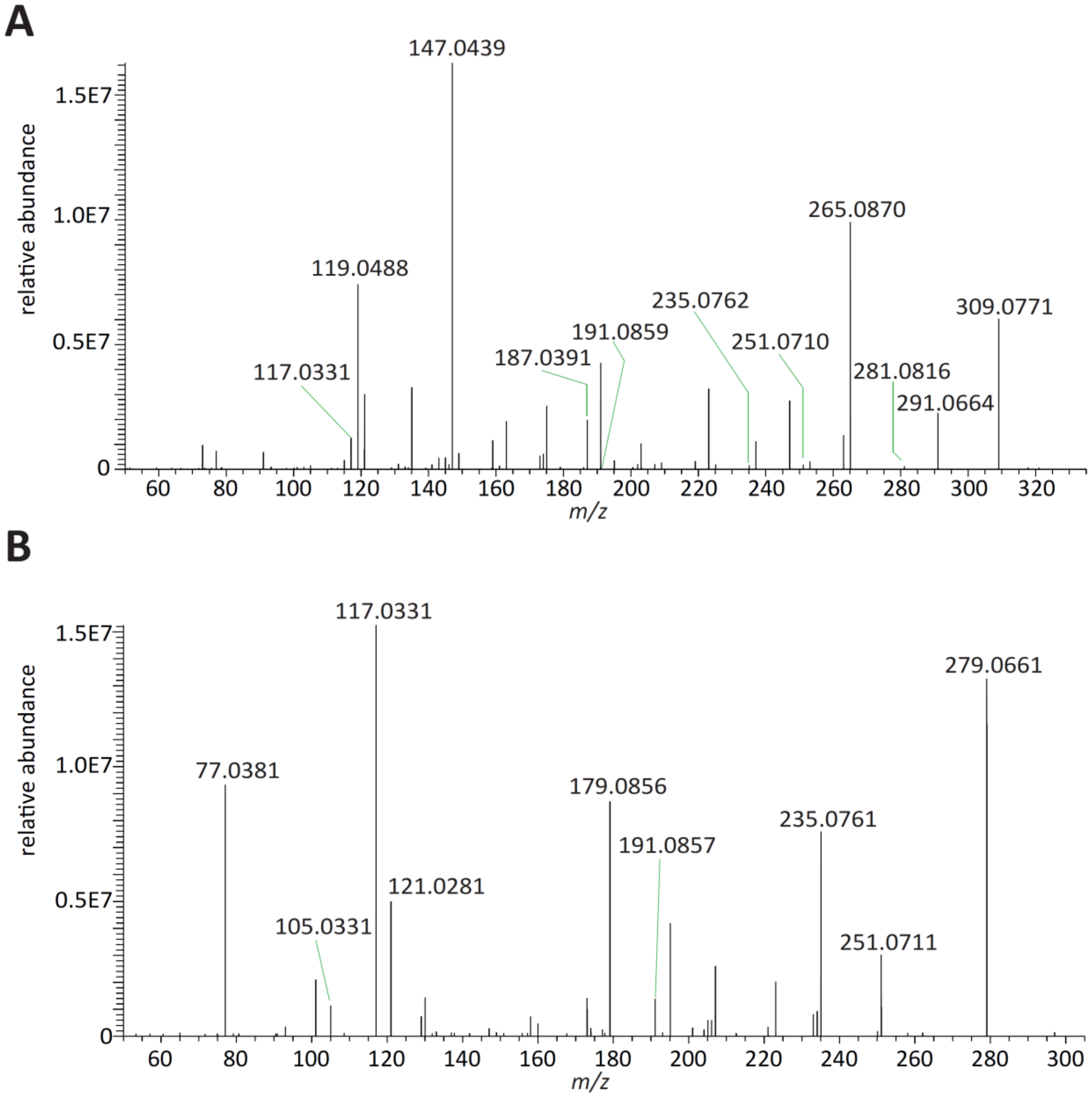

Figure S13. MS/MS fragmentation of major compounds produced by *A. niger* tPS11. (**A**) phlebiopsin A; (**B**) phlebiopsin B. The spectra were recorded in negative ionization mode.

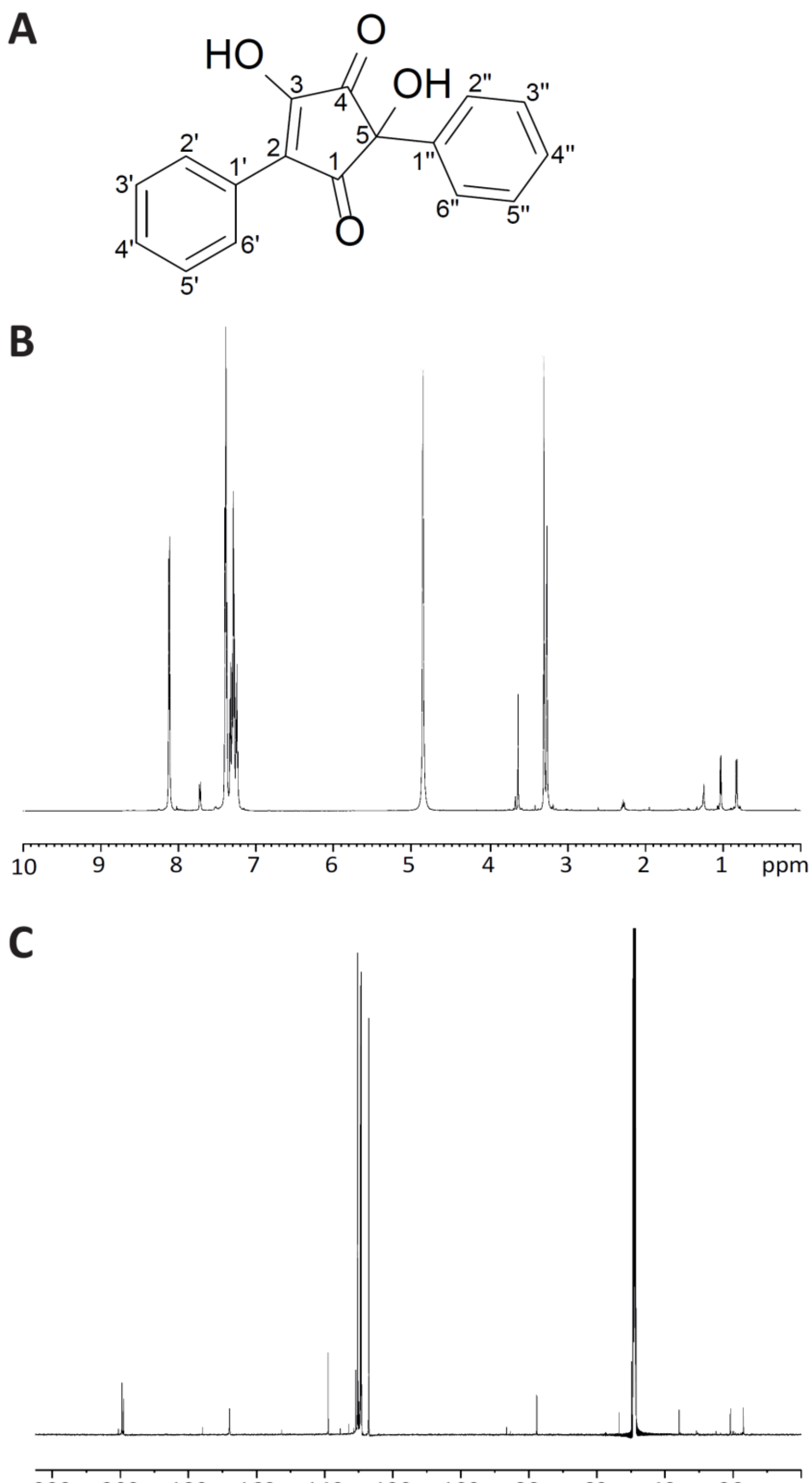

Figure S14. 1D NMR spectra of phlebiopsin B. The compound was purified from *A. niger* tPS11 to > 90% purity. (**A**) Structure of phlebiopsin B**.** (**B**) ^{1}H and (**C**) ^{13}C NMR spectrum (600 MHz for ^{1}H, 150 MHz for ^{13}C, CD_3OD, 300 K).

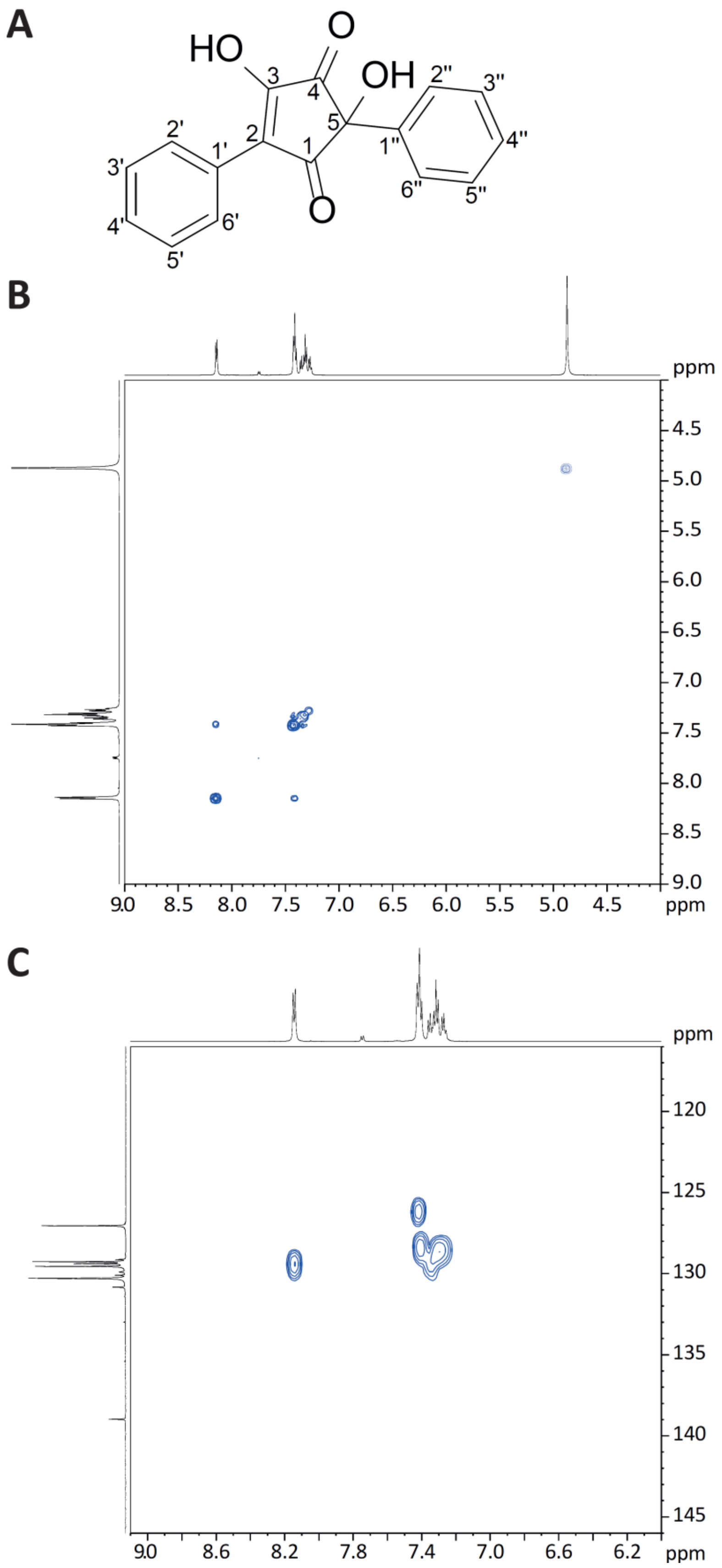

Figure S15. 2D NMR spectra of phlebiopsin B. The compound was purified from *A. niger* tPS11 to > 90% purity. (**A**) Structure of phlebiopsin B**.** (**B**) ^{1}H,^{1}H COSY and (**C**) ^{1}H,^{13}C HSQC spectrum (CD_3OD, 300 K).

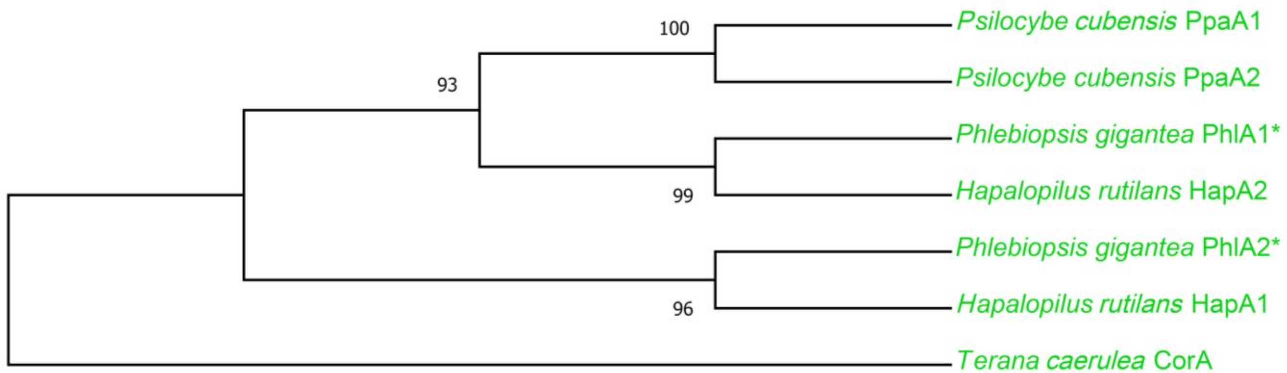

Figure S16. Phylogenetic analysis of clade I quinone synthetases. The analysis is based on the respective adenylation domains. The A domains were aligned using ClustalW2 [1] implemented in the MEGA X software [2].The evolutionary history was inferred by using the Maximum Likelihood method and Le_Gascuel_2008 model [3]. The bootstrap consensus tree inferred from 1000 replicates is taken to represent the evolutionary history of the taxa analyzed [4]. Branches corresponding to partitions reproduced in less than 50% bootstrap replicates are collapsed. The percentage of replicate trees in which the associated taxa clustered together in the bootstrap test (1000 replicates) are shown next to the branches [4]. Initial tree(s) for the heuristic search were obtained automatically by applying Neighbor-Join and BioNJ algorithms to a matrix of pairwise distances estimated using the JTT model [5], and then selecting the topology with superior log likelihood value. A discrete Gamma distribution was used to model evolutionary rate differences among sites (5 categories (+G, parameter = 0.9383)). This analysis involved 7 amino acid sequences. All positions with less than 90% site coverage were eliminated, i.e., fewer than 10% alignment gaps, missing data, and ambiguous bases were allowed at any position (partial deletion option). There was a total of 525 positions in the final dataset. Evolutionary analyses were conducted in MEGA X [2]. * The *Phlebiopsis gigantea* genome (Genbank: AZAG00000000.1 [6]) was browsed for *corA*-like genes (Genbank: OM515349.1 [7]). Two genetic loci, PHLGIDRAFT_36214 and PHLGIDRAFT_229718 were identified, referred to as *phlA1* and *phlA2*, respectively. The corresponding enzymes PhlA1 (Protein ID: KIP05892.1) and PhlA2 (Protein ID: KIP03659.1) are hypothetical and have not been characterized experimentally.

References

1. Larkin MA, Blackshields G, Brown NP, Chenna R, McGettigan PA, McWilliam H, Valentin F, Wallace IM, Wilm A, Lopez R *et al*: **Clustal W and Clustal X version 2.0**. *Bioinformatics* 2007, **23**(21):2947-2948.
2. Kumar S, Stecher G, Li M, Knyaz C, Tamura K: **MEGA X: Molecular Evolutionary Genetics Analysis across Computing Platforms**. *Mol Biol Evol* 2018, **35**(6):1547-1549.
3. Le SQ, Gascuel O: **An improved general amino acid replacement matrix**. *Mol Biol Evol* 2008, **25**(7):1307-1320.
4. Felsenstein J: **Confidence Limits on Phylogenies: An Approach Using the Bootstrap**. *Evolution* 1985, **39**(4):783-791.
5. Jones DT, Taylor WR, Thornton JM: **The rapid generation of mutation data matrices from protein sequences**. *Comput Appl Biosci* 1992, **8**(3):275-282.
6. Hori C, Ishida T, Igarashi K, Samejima M, Suzuki H, Master E, Ferreira P, Ruiz-Dueñas FJ, Held B, Canessa P: **Analysis of the *Phlebiopsis gigantea* genome, transcriptome and secretome provides insight into its pioneer colonization strategies of wood**. *PLoS Genetics* 2014, **10**(12):e1004759.
7. Lawrinowitz S, Wurlitzer JM, Weiss D, Arndt HD, Kothe E, Gressler M, Hoffmeister D: **Blue Light-Dependent Pre-mRNA Splicing Controls Pigment Biosynthesis in the Mushroom *Terana caerulea***. *Microbiol Spectr* 2022, **10**(5):e0106522.

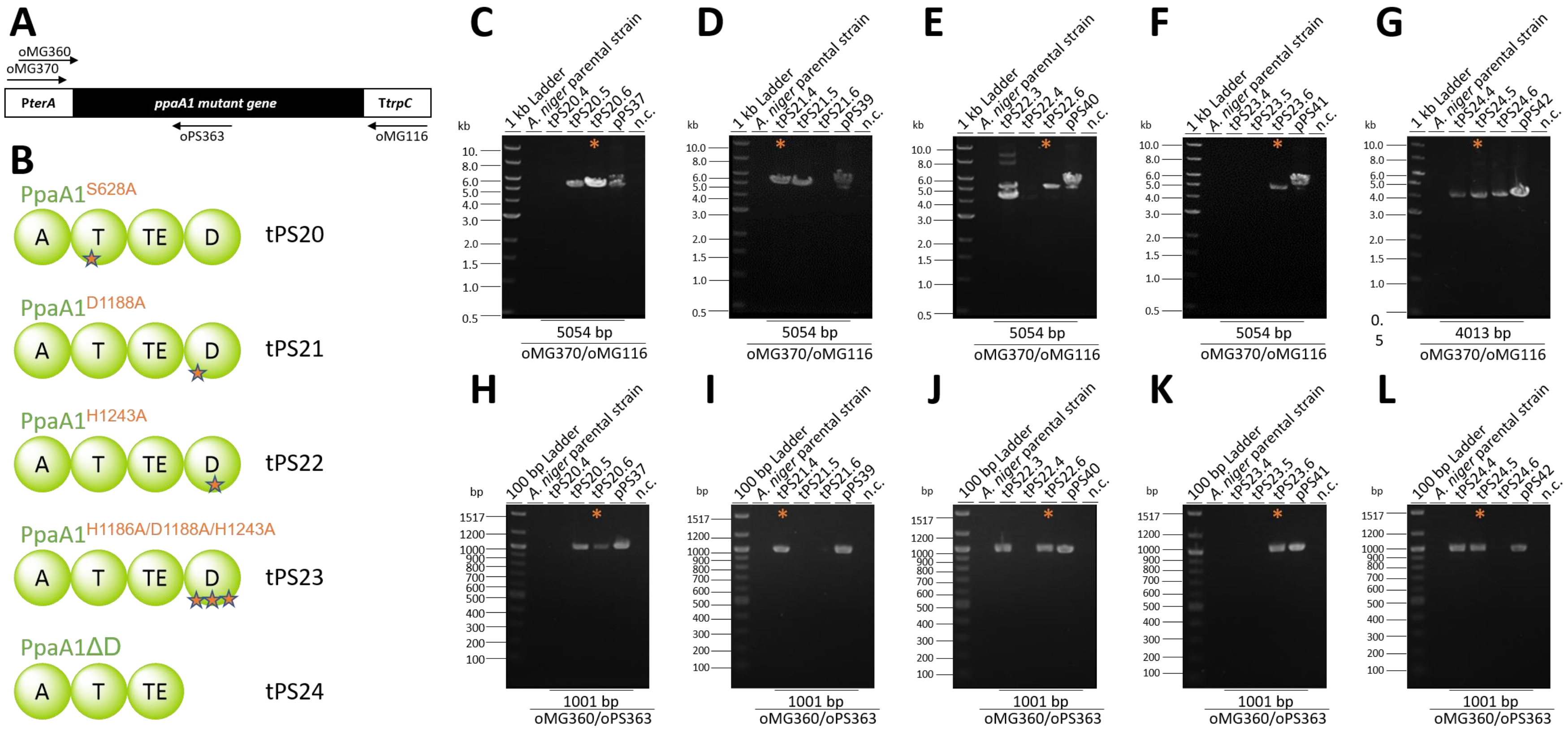

Figure S17. PCR to verify *P. cubensis ppaA1* mutant gene integration into the *A. niger* genome. (**A**) Schematic representation of the oligonucleotide binding sites of oMG360 and oMG370 in P*alcA*, oPS363 in *ppaA1* and oMG116 in T*trpC.* Full length integration in the *A. niger* host genome was verified by PCR using oligonucleotide pairs oMG370/oMG116 (binding in P*alcA* and T*trpC.* Moreover, integration of *ppaA1* mutant constructs were verified by PCR using oligonucleotide pairs oMG360/oPS363 (binding in P*alcA* and *ppaA1*). (**B**) Schematic representation of domain structures. Orange stars indicate a mutation. (**C-G**) Verification of full-length integration of: (**C**) *ppaA1*_S628A; (**D**) *ppaA1*_D1188A; (**E**) *ppaA1*_H1243A; (**F**) *ppaA1*_H1186A/D1188A/H1243A; (**G**) *ppaA1*ΔD. The integration of *ppaA1* mutant constructs into the *A. niger* genome were verified by amplification of a gene specific partial fragment: (**H**) *ppaA1*_S628A. (**I**) *ppaA1*_D1188A. (**J**) *ppaA1*_H1243A. (**K**) *ppaA1*_H1186A/D1188A/H1243A. (**L**) *ppaA1*ΔD. In each case, dH_2O (n.c.) and gDNA of the *A. niger* ATNTΔ*pyrG*24x parental strain served as negative controls. DNA of plasmids pPS37 (encoding *ppaA1*_S628A, panels **C/H**), pPS39 (encoding *ppaA1*_D1188A, panels **D/I**), pPS40 (encoding *ppaA1*_H1243A, panels **E/J**), pPS41 (encoding the triple mutant gene *ppaA1*_H1186A/D1188A/H1243A, panels **F/K**), and pPS42 (encoding the *ppaA1*ΔD-domain, panels **G/L**) were used as positive controls. PCR amplicons were visualized in ethidium bromide-stained agarose gels. Orange asterisks indicate the transformants chosen for further work.

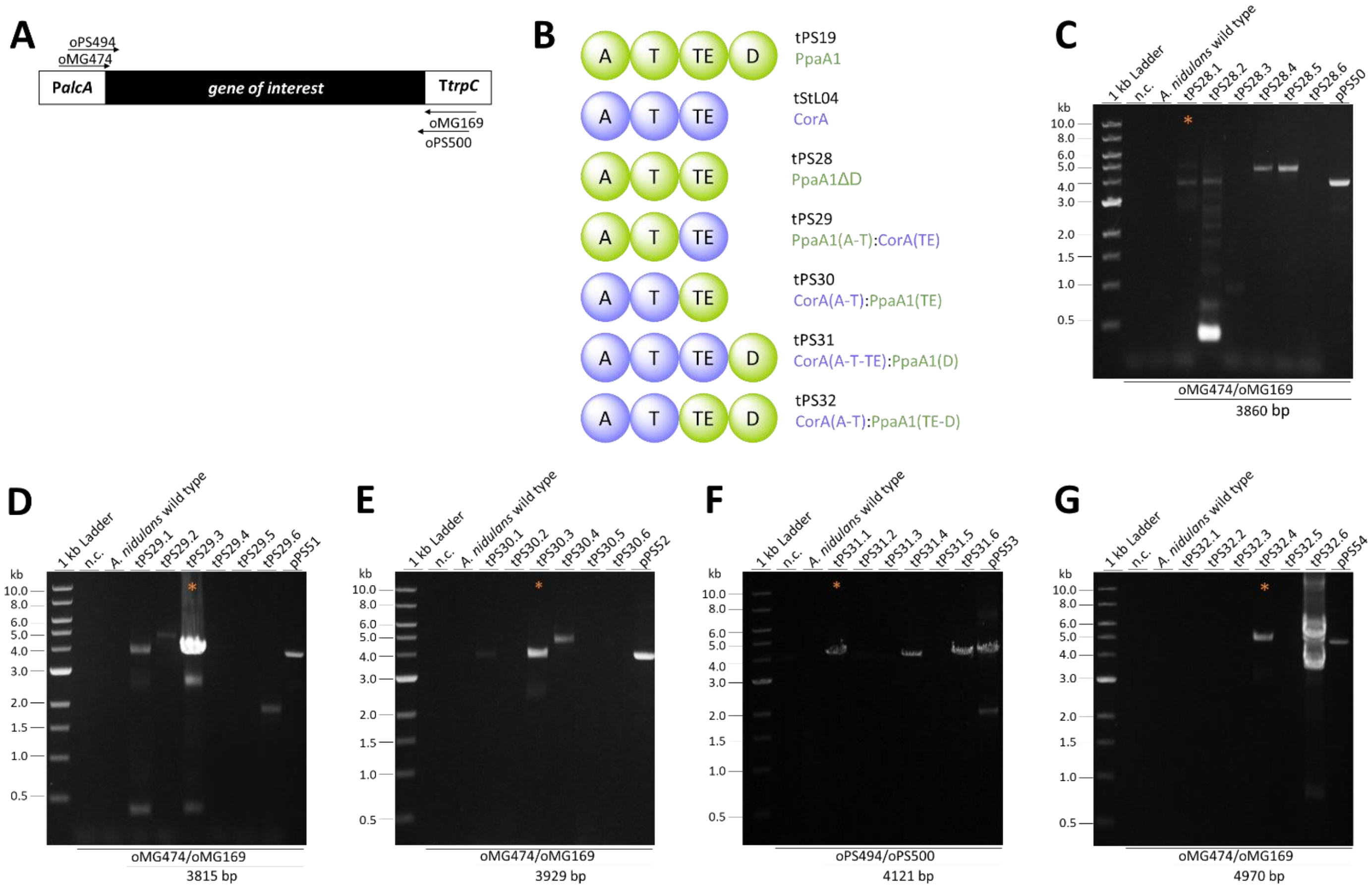

Figure S18. PCR to verify *ppaA1/corA* hybrid gene integration into the *A. nidulans* genome. (**A**) Schematic representation of oligonucleotide binding sites of oMG474 and oPS494 in P*alcA* and oMG169 and oPS500 in T*trpC*. Integration of *ppaA1/corA* hybrids in the *A. nidulans* genome was verified by PCR using oligonucleotide pairs oMG474/oMG169 or oPS494/oPS500 (binding in P*alcA* and T*trpC*). (**B**) Domain structure of the mutants (green for PpaA1, violet for CorA). dH_2O (n.c.) and gDNA of *A. nidulans* FGSC A4 (wild type) served as negative controls. DNA of plasmids pPS50 (encoding *ppaA1*ΔD, panel **C**), pPS51 (encoding *ppaA1(A-T)::corA(TE)*, panel **D**), pPS52(encoding *corA(A-T)::ppaA1(TE)*, panel **E**), pPS53 (encoding *corA(A-T-TE)::ppaA1(D),* panel **F**) and pPS54 (encoding *corA(A-T)::ppaA1(TE-D),* panel **G**) were used as positive controls. PCR amplicons were visualized in ethidium bromide-stained agarose gels. Orange asterisks indicate the transformants chosen for further work.

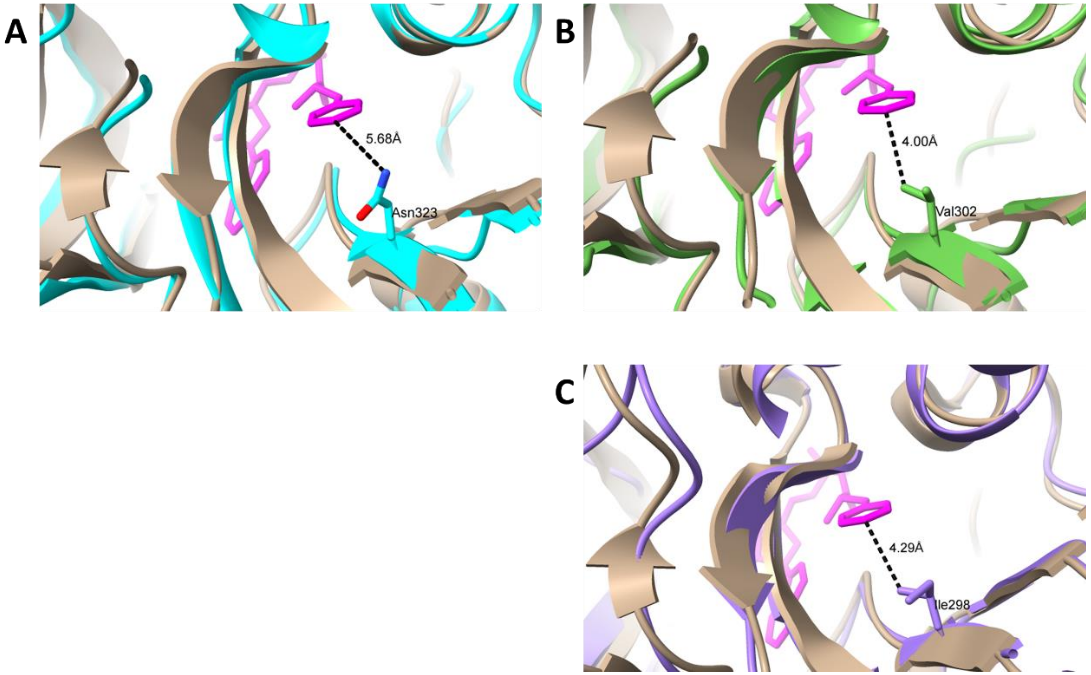

Figure S19. Structure prediction of quinone synthetases. The models of (**A**) *S. lacrymans* NPS3 (cyan), (**B**) *P. cubensis* PpaA1 (green) and (**C**) *T. caerulea* CorA (violet) were predicted with AlphaFold2 [1]. ChimeraX software [2, 3] was used to superimpose each of the models with the experimentally verified structure of the McyG A-PCP didomain (PDBe 4r0m [4]; brown) co-crystallized with phenylalanyl-adenylate (magenta). Panels A-C show close-up views of a portion of the adenylation domain around the specificity-conferring position identified in our study. The structures of the respective amino acid residues (Figure 8 and Figure S1) are shown (Asn323 of NPS3, Val302 of PpaA1 and Ile298 of CorA). The oxygen and nitrogen atoms of the carboxylic acid amide group of Asn323 are marked in red and blue, respectively (panel A). The distance between C4 of the substrate phenyl ring and the amino acid residue co-conferring specificity was calculated using ChimeraX [2, 3].

References

1. Mirdita M, Schütze K, Moriwaki Y, Heo L, Ovchinnikov S, Steinegger M: **ColabFold: making protein folding accessible to all**. *Nat Methods* 2022, **19**(6):679-682.
2. Goddard TD, Huang CC, Meng EC, Pettersen EF, Couch GS, Morris JH, Ferrin TE: **UCSF ChimeraX: Meeting modern challenges in visualization and analysis**. *Protein Sci* 2018, **27**(1):14-25.
3. Pettersen EF, Goddard TD, Huang CC, Meng EC, Couch GS, Croll TI, Morris JH, Ferrin TE: **UCSF ChimeraX: Structure visualization for researchers, educators, and developers**. *Protein Sci* 2021, **30**(1):70-82.
4. Tan XF, Dai YN, Zhou K, Jiang YL, Ren YM, Chen Y, Zhou CZ: **Structure of the adenylation-peptidyl carrier protein didomain of the *Microcystis aeruginosa* microcystin synthetase McyG**. *Acta Crystallogr D Biol Crystallogr* 2015, **71**(Pt 4):873-881.

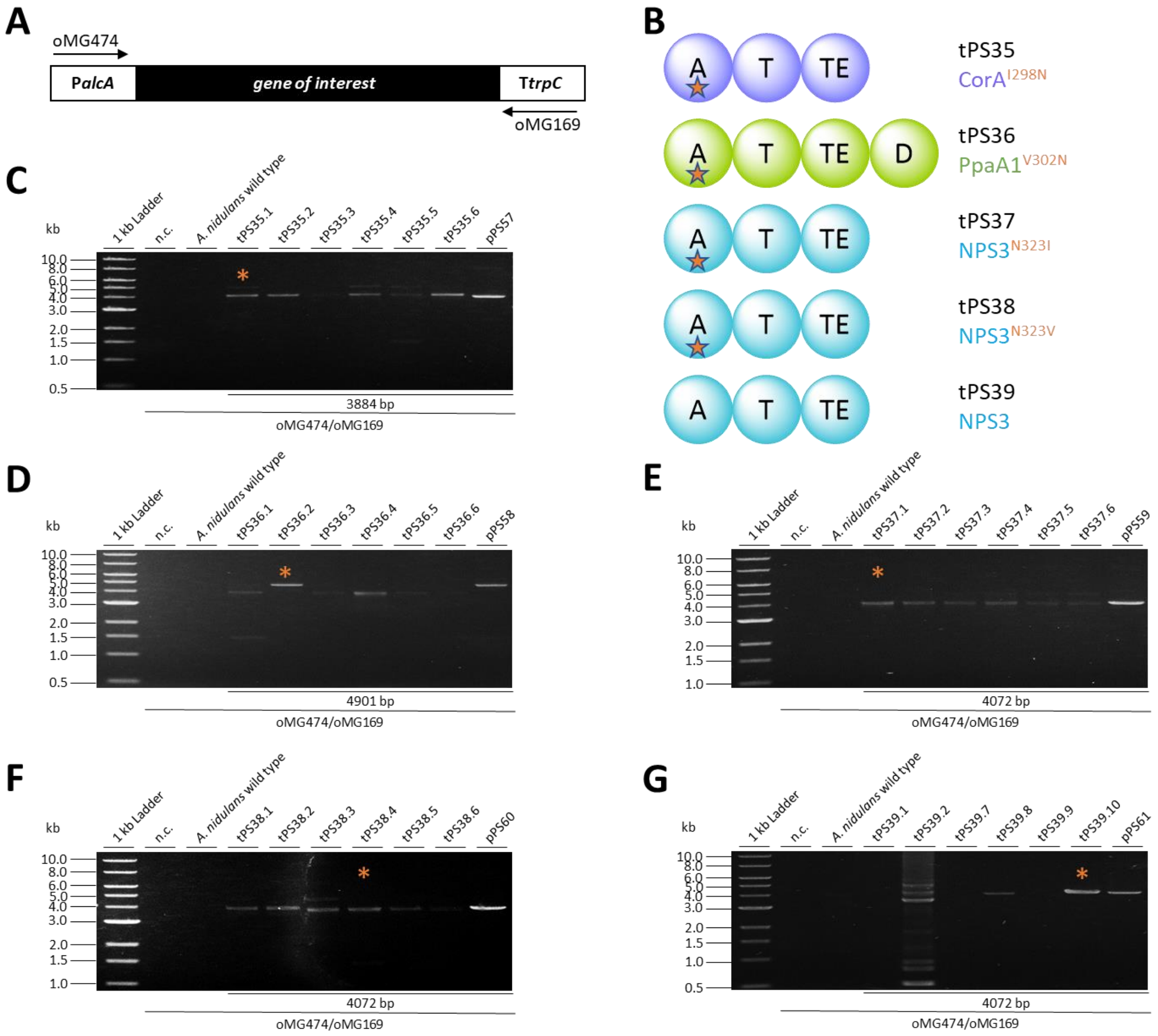

Figure S20. PCR to verify *corA*-, *ppaA1*-, *nps3*-mutant and native *nps3* integration into the *A. nidulans* genome. (**A**) Schematic representation of oligonucleotide binding sites of oMG474 in P*alcA* and oMG169 in T*trpC*. Integration of *corA, ppaA1* and *nps3* mutants as well as native *nps3* in the *A. nidulans* genome was verified by PCR using oligonucleotide pairs oMG474/oMG169 (targeting P*alcA* and T*trpC*). (**B**) Schematic representation of domain structures of quinone synthetases (green for PpaA1, violet for CorA, orange for Nps3). Orange stars indicate a mutation. (**C**) Verification of the full-length integration of *corA*_I298N. DNA of plasmid pPS57 (encoding *corA*_I298N) served as positive control. (**D**) Verification of the full-length integration of *ppaA1*_V302N. DNA of plasmid pPS58 (encoding *ppaA1*_V302N) served as positive control. (**E**) Verification of the full-length integration of *nps3*_N323I. DNA of plasmid pPS59 (encoding *nps3*_N323I) served as positive control. (**F**) Verification of the full-length integration of *nps3*_N323V. DNA of plasmid pPS60 (encoding *nps3*_N323V) served as positive control. (**G**) Verification of the full-length integration of *nps3.* DNA of plasmid pPS61 (encoding *nps3*) was used as positive control. In each case, dH_2O (n.c.) and gDNA of the *A. nidulans* FGSC A4 parental strain (wild type) served as negative controls. DNA of plasmids PCR amplicons were visualized in ethidium bromide-stained agarose gels. Orange asterisks indicate the transformants chosen for further work.

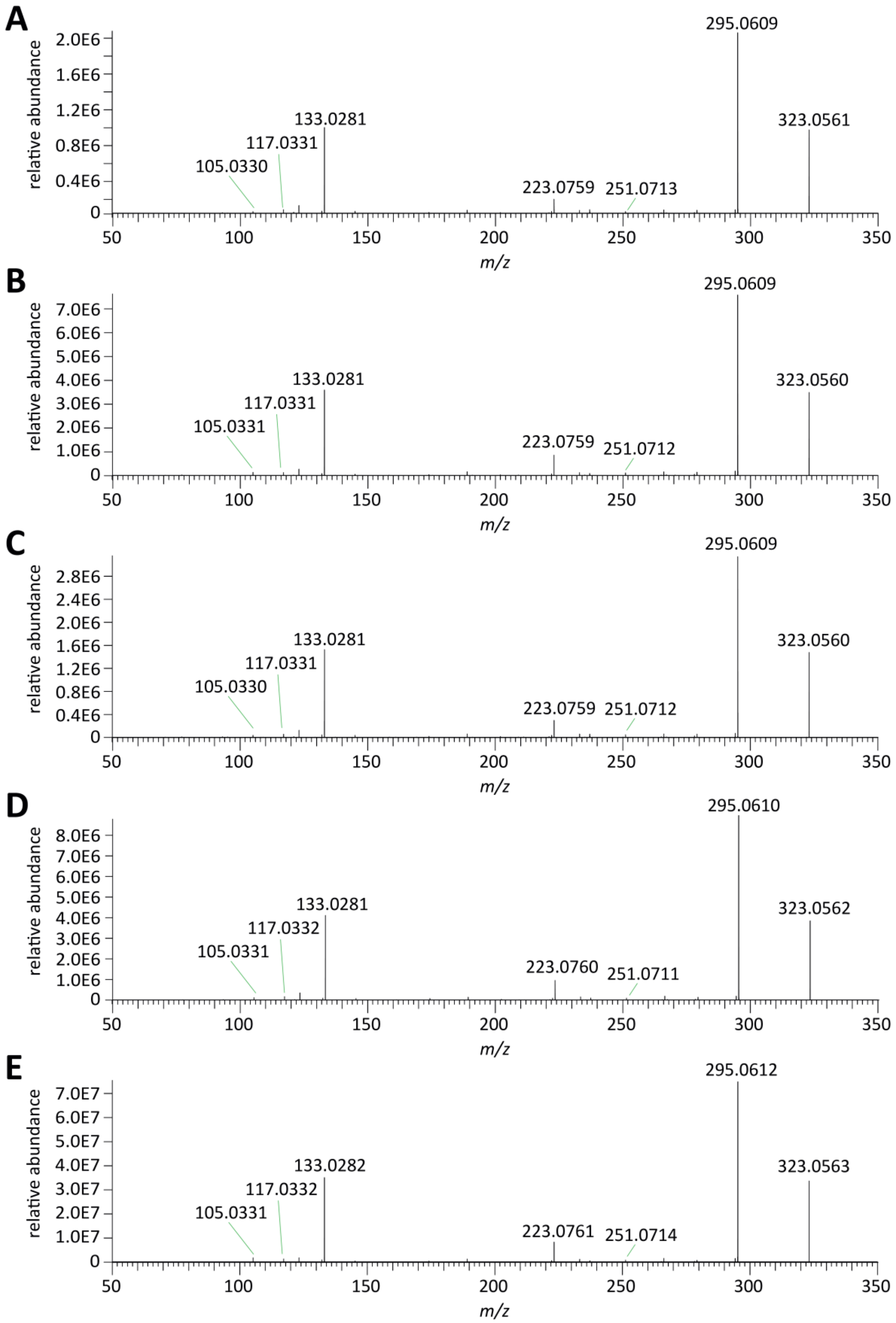

Figure S21. MS/MS fragmentation of atromentin, produced *in vivo*. Atromentin produced (**A**) by *A. nidulans* tPS36; (**B**) by *A. nidulans* tPS37; (**C**) by *A. nidulans* tPS38; (**D**) by *A. nidulans* tPS39. (**E**) MS/MS spectrum of an atromentin standard. The spectra were recorded in negative ionization mode.

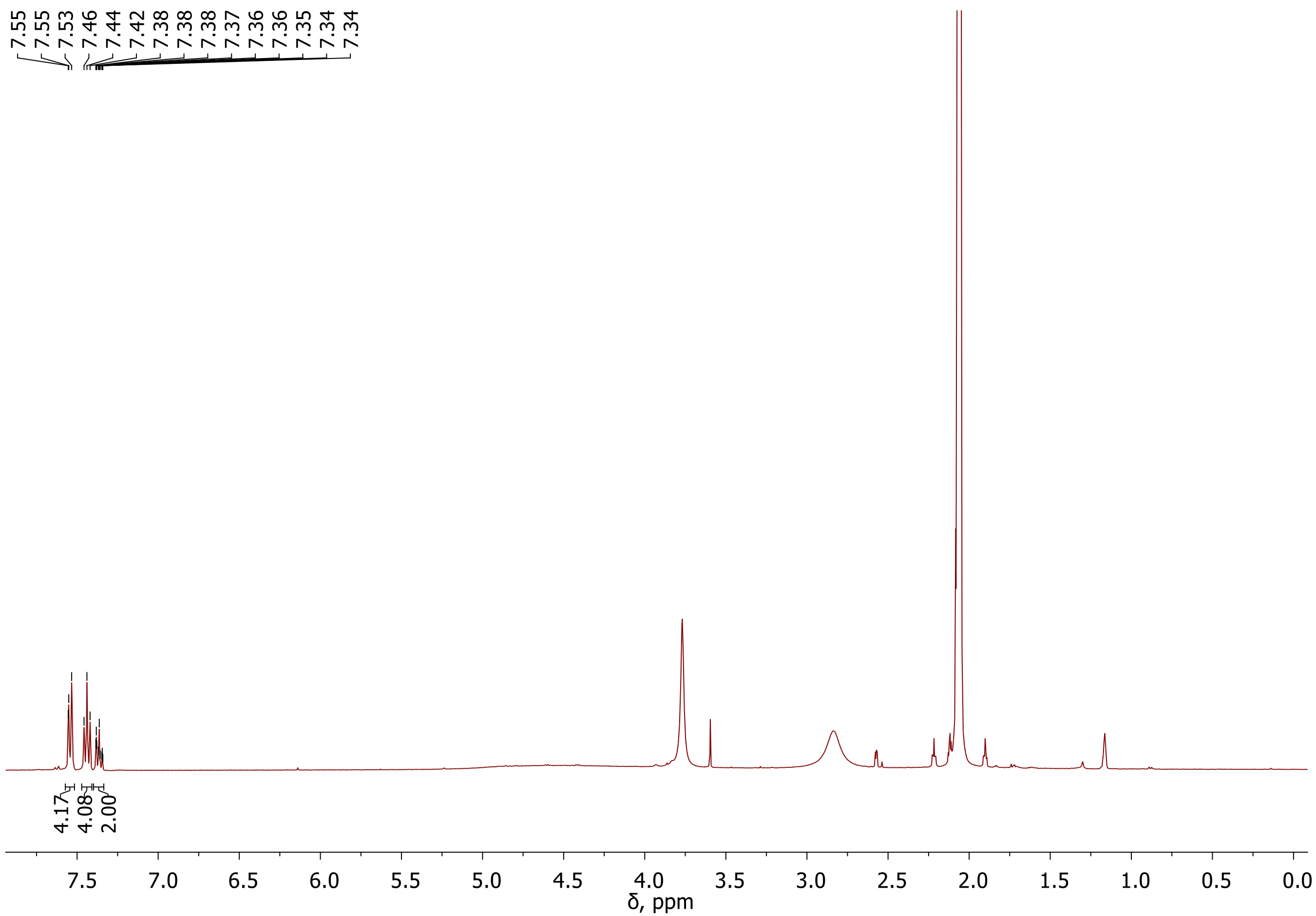

Figure S22. ^{1}H NMR spectrum of synthesized 2,5-dihydroxy-3,6-diphenyl-1,4-benzoquinone (polyporic acid). 400 MHz, $(CD_3)_2CO$.

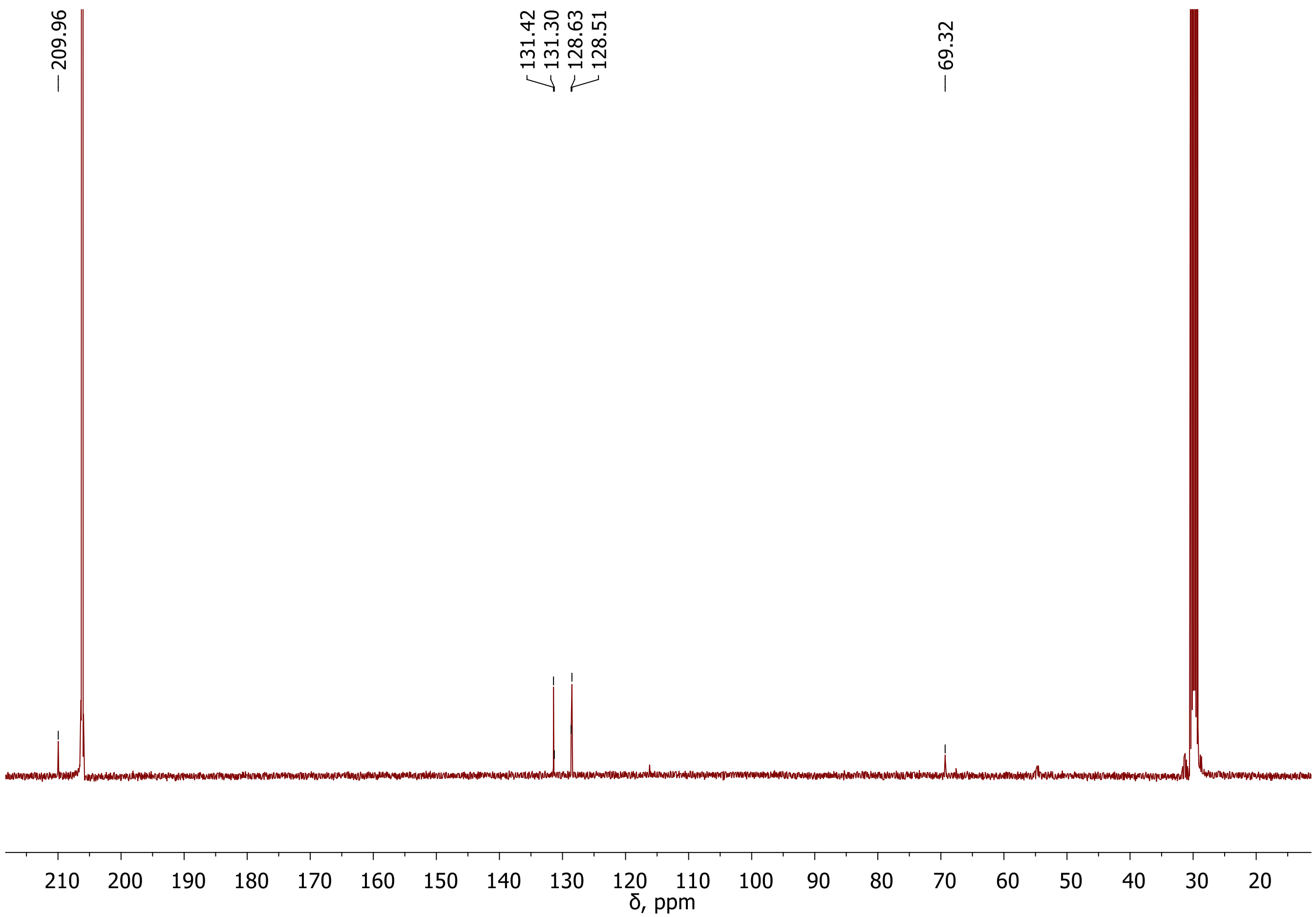

Figure S23. ^{13}C NMR spectrum of synthesized 2,5-dihydroxy-3,6-diphenyl-1,4-benzoquinone (polyporic acid). 101 MHz, $(CD_3)_2CO$.
