# Supplementary material for: Bifurcate evolution of quinone synthetases in basidiomycetes
Source: Fungal Biol Biotechnol. 2023 Jul 3;10:14. doi: 10.1186/s40694-023-00162-1 (PMC10316625; doi:10.1186/s40694-023-00162-1)
Supplement: Supplementary file 3 — Additional file 3. Experimental procedures. [file 40694_2023_162_MOESM3_ESM.pdf]

Experimental procedures

General experimental procedures. Chemicals, media ingredients, and solvents were purchased from Carl Roth, Sigma-Aldrich, and VWR. Oligonucleotides were synthesized by Integrated DNA Technologies and are listed in Additional file 1: Tables S7 and S9. Restriction enzymes were procured from NEB.

After linearization of vectors, their ends were dephosphorylated using Quick CIP phosphatase (NEB). DNA gel extraction, restriction, dephosphorylation, ligation, and plasmid isolation followed the manufacturers' instructions (Macherey-Nagel, NEB). For all expression plasmids (Additional file 1: Table S4), correct assembly of inserts with vectors was verified by colony PCR (Additional file 1: Table S8, PCR method D), analytical restriction digests and DNA sequencing (GENEWIZ Inc.).

Phylogentic analysis of *Terana caerulea* CorA and related quinone synthetases based on the respective adenylation domains. The evolutionary history of the quinone synthetase A domains was inferred by using the Maximum Likelihood method and Le_Gascuel_2008 model [1]. The bootstrap consensus tree inferred from 1000 replicates was taken to represent the evolutionary history of the analyzed taxa [2]. Branches corresponding to partitions reproduced in less than 50% bootstrap replicates were collapsed. Initial tree(s) for the heuristic search were obtained automatically by applying Neighbor-Join and BioNJ algorithms to a matrix of pairwise distances estimated using the JTT model [3], and then selecting the topology with superior log likelihood value. A discrete Gamma distribution was used to model evolutionary rate differences among sites (5 categories (+G, parameter = 1.6206)). The rate variation model allowed for some sites to be evolutionarily invariable ([+I], 4.87% sites). This analysis involved 21 amino acid sequences. All positions with less than 90% site coverage were eliminated, i.e., fewer than 10% alignment gaps, missing data, and ambiguous bases were allowed at any position (partial deletion option). The final dataset included a total of 493 positions. Evolutionary analyses were conducted in MEGA X [4].

Construction of plasmids for heterologous expression of *hapA1* and *hapA2*. The *hapA* genes were amplified from genomic DNA (Additional file 1: Table S8, PCR method B) using the oligonucleotides oSS189/190 (*hapA1*) and oSS191/192 (*hapA2*). The gel-purified fragments were ligated to pUC19, amplified from plasmid pMG49, using oligonucleotides oMG468 and oMG469. The PCR product was treated with *Dpn*I (NEB) to remove methylated DNA template and subsequently used to construct expression plasmids pSS10 (chromosomal *hapA1*)

and pSS11 (chromosomal *hapA2*). Inserts and vector backbone were ligated by the Gibson assembly strategy using NEBuilder HiFi DNA Assembly Cloning Kit (NEB).

Construction of *A. nidulans* expression vector pPS29. Plasmid pMG49 [5] served as template to amplify two fragments (Additional file 1: Table S8, PCR method E) using the oligonucleotides oPS441/438 and oPS439/442. The gel-purified fragments were assembled using the NEBuilder HiFi DNA Assembly Cloning Kit (NEB) to create pPS29. Thus, the sequence **ATG***CGATCGCGA*CATCATCACCATCACCAT**TAG** was introduced, including an **ATG** start and a **TAG** stop codon, *Asi*SI (*GCGATCGC*) and *Nru*I (*TCGCGA*) restriction sites as well as codons for a hexahistidine tag (CATCATCACCATCACCAT).

Construction of plasmids for heterologous expression of *ppaA1* and *ppaA2*. The coding sequences were PCR amplified from cDNA using the oligonucleotides oPS355/356 (*ppaA2*) and oPS357/358 (*ppaA1*) (Additional file 1: Table S8, PCR method C). The gel-purified fragments were ligated to the *Nco*I-restricted plasmid pSMX2-URA [6] using the NEBuilder HiFi DNA Assembly Cloning Kit (NEB) to yield expression plasmids pPS13 (*ppaA2*) and pPS14 (*ppaA1*). The coding sequences were also PCR-amplified from cDNA using the oligonucleotides oPS429/430 (*ppaA2*) and oPS431/432 (*ppaA1*) (Additional file 1: Table S8, PCR method C). The gel-purified fragments were ligated into the *Asi*SI-restricted plasmid pPS29 using the NEBuilder HiFi DNA Assembly Cloning Kit (NEB) to yield expression plasmids pPS35 (*ppaA2*) pPS36 (*ppaA1*).

Construction of plasmids harboring *ppaA1* mutants to transform *A. niger*. Plasmid pPS14 was used as template to amplify PCR fragments in order to mutate *ppaA1*. Two or three oligonucleotide pairs each were used to introduce the respective mutations (Additional file 1: Table S4 and Table S9): oPS459/462 and oPS460/461 to construct pPS37, oPS459/466 and oPS460/465 to construct pPS39, oPS459/468 and oPS460/467 to construct pPS40, oPS459/470, oPS460/467 and oPS468/469 to construct pPS41. The corresponding fragments were assembled with NEBuilder HiFi DNA Assembly Cloning Kit (NEB). For a deletion of the dioxygenase domain, cDNA was used as PCR template together with oligonucleotides oPS357/471 to amplify an insert that was assembled with *Nco*I restricted plasmid pSMX2-URA [6] to yield plasmid pPS42.

Construction of *A. nidulans* expression plasmids for *ppaA1*ΔD and hybrid *ppaA1*/*corA* genes. Expression plasmid pPS50 was constructed to produce an PpaA1 version lacking the C-terminal dioxygenase domain. To amplify the altered *ppaA1* gene, pPS14 was used as template and oligonucleotides oPS431/490 as primers for PCR (Additional file 1: Table S8, PCR method C). For *ppaA1*/*corA* domain swaps, pPS14 (encoding *ppaA1*) and pSS06 (encoding *corA*) [7] served as templates. The oligonucleotides oPS431/491 (hybridizing with *ppaA1*) and oPS492/493 (hybridizing with *corA*) were used to amplify two PCR fragments (Additional file 1: Table S8, PCR method C). The gel purified fragments were assembled with *Asi*SI-restricted plasmid pPS29 using the NEBuilder HiFi DNA Assembly Cloning Kit (NEB) to construct pPS51. Analogously, two couples of oligonucleotides (each) were used to amplify PCR fragments to construct pPS52, pPS53, and pPS54, respectively: oPS494/495 (hybridizing with *corA*) and oPS496/497 (hybridizing with *ppaA1*); oPS494/499 (hybridizing with *corA*) and oPS499/500 (hybridizing with *ppaA1*); oPS494/495 (hybridizing with *corA*) and oPS496/500 (hybridizing with *ppaA1*). Corresponding fragments were assembled with *Asi*SI-restricted plasmid pPS29 using the NEBuilder HiFi DNA Assembly Cloning Kit (NEB).

Construction of plasmids to express mutated quinone synthetase genes in *A. nidulans*. To replace an isoleucin with a asparagine codon (I298N) in *corA* and to construct pPS57*,* plasmid pSS06, and oligonucleotides oPS494/575 and oPS574/493 were used for PCR. Plasmid pPS36 and oligonucleotides oPS431/583 and oPS582/432 were used to amplify PCR fragments for *ppaA1* mutation V302N, inserted in plasmid pPS58. Genomic DNA from *Serpula lacrymans* served as template to amplify plasmid inserts for mutated *nps3* versions. Oligonucleotides oPS576/579 and oPS578/577 (to create pPS59) as well as oPS576/581 and oPS580/577 (to create pPS60) were used to introduce the mutations N323I and N323V, respectively. In each case, the corresponding inserts (all amplified using PCR method C, Additional file 1: Table S8) were ligated to the *Asi*SI-restricted plasmid pPS29, using the NEBuilder HiFi DNA Assembly Cloning Kit (NEB).

Construction of a plasmid for heterologous expression of *nps3* in *A. nidulans*. The *nps3* coding sequence was amplified from genomic DNA, using oligonucleotides oPS576/577 and PCR method C (Additional file 1: Table S8). The insert and the *Asi*SI restricted plasmid pPS29 were asembled using the NEBuilder HiFi DNA Assembly Cloning Kit (NEB) to yield *nps3* expression plasmid pPS61.

PCR-based genotyping of *Aspergillus* transformants. PCR method A (Additional file 1: Table S8) was applied for genotyping of tPS11, tPS15 and tPS18-24 (Additional file 1: Table S6, Additional file 2: Fig. S8-S10 and S17). Genotyping of tStL07 and tStL08 (Additional file 1: Table S6, Additional file2: Fig. S2) was performed using PCR method B (Additional file 1: Table S8). PCR method C (Additional file 1: Table S8) was applied to genotypically characterize tPS10, tPS28-32 and tPS35-39 (Additional file 1: Table S6, Additional file 2: Fig. S9, S18 and S20).

Chemical synthesis of polyporic acid. To a mixture of 2,5-dihydroxy-1,4-benzoquinone (5.20 g, 37.1 mmol, 1 equiv.) and dry MeOH (80 mL) $BF_3{\cdot}Et_2O$ (13.0 mL, 105 mmol, 2.85 equiv.) was added under a nitrogen atmosphere. The resulting suspension was stirred at 70°C for 2 h. The solid was filtered, washed with cooled MeOH and dried under reduced pressure (0.05 mbar) to give 2,5-dimethoxy-1,4-benzoquinone (5.20 g, 83%) as a yellow solid. N-Bromosuccinimide (5.80 g, 32.6 mmol, 2 equiv.) was slowly added to a mixture of 2,5 dimethoxy-1,4-benzoquinone (2.71 g, 16.1 mmol, 1 equiv.) and dry DMF (80 mL) under a nitrogen atmosphere. The resulting suspension was stirred for 16 h, diluted with water (160 mL) and extracted with EtOAc (3 × 80 mL). Combined organic extracts were dried over Na_2SO_4 and concentrated under reduced pressure. The residue was purified via silica gel column chromatography (200 g, CH_2Cl_2-hexanes, 2:3) giving 2,5 dibromo-3,6-dimethoxy-1,4-benzoquinone (4.43 g, 84%) as a red crystalline solid.

A mixture of 2,5-dibromo-3,6-dimethoxy-1,4-benzoquinone (326 mg, 1.00 mmol, 1 equiv.), dichloro-bis(triphenylphosphine)-palladium (II) (70 mg, 0.10 mmol, 0.1 equiv.), K_2CO_3 (690 mg, 5.00 mmol, 5 equiv.), phenylboronic acid (488 mg, 4.00 mmol, 4 equiv.), and dry 1,4-dioxane (10 mL) was stirred for 24 h in a Carius tube at 110°C. The reaction mixture was cooled to room temperature, diluted with CH_2Cl_2 (10 mL), and filtered through a Celite pad. The filtrate was concentrated under reduced pressure. The residue was purified via silica gel column chromatography (50 g, CH_2Cl_2-hexanes, 3:2) giving 2,5-dimethoxy-3,6-diphenyl-1,4-benzoquinone (150 mg, 47%) as an orange solid.

To a solution of 2,5-dimethoxy-3,6-diphenyl-1,4-benzoquinone (2.00 g, 6.25 mmol, 1 equiv.) in dry CH_2Cl_2 (200 mL) BBr_3 (1 M in CH_2Cl_2, 26.1 mL, 26.1 mmol, 4.2 equiv.) was added at –78°C under a nitrogen atmosphere. The resulting solution was stirred for 16 h while adjusting to room temperature. MeOH (10 mL) was added, and the resulting mixture concentrated under reduced pressure. The residue was recrystallized from 1,4-dioxane (150 mL) to give 2,5-dihydroxy-3,6-diphenyl-1,4-benzoquinone (1.75 g, 96%) as a dark brown solid.

Melting point: 258-260°C (subl.).

TLC: R_f = 0.10 (CH_2Cl_2).

^{1}H NMR: (400 MHz, $(CD_3)_2CO$) δ = 7.57 – 7.52 (m, 4H, Ph, 4 × Ph 2-H), 7.44 (t, J = 7.4 Hz, 4H, 4 × Ph 3-H), 7.40 – 7.33 (m, 2H, 2 × Ph 4-H) ppm.

^{13}C NMR: (101 MHz, $(CD_3)_2CO$) δ = 210.0, 131.4, 131.3, 128.6, 128.5, 69.3 ppm.

IR (ATR)**:** $\tilde{\nu}$ = 3306 (s), 2361 (s), 2342 (s), 1611 (s), 1323 (m), 1308 (s), 1244 (s), 993 (s), 692 (m) cm^{-1}.

HRMS (ESI)**:** *m/z* $[M+H]^+$ calculated for $C_{18}H_{12}O_4$ 293.0809; found: 293.0812.

References

1. Le SQ, Gascuel O: **An improved general amino acid replacement matrix**. *Mol Biol Evol* 2008, **25**(7):1307-1320.
2. Felsenstein J: **Confidence Limits on Phylogenies: An Approach Using the Bootstrap**. *Evolution* 1985, **39**(4):783-791.
3. Jones DT, Taylor WR, Thornton JM: **The rapid generation of mutation data matrices from protein sequences**. *Comput Appl Biosci* 1992, **8**(3):275-282.
4. Kumar S, Stecher G, Li M, Knyaz C, Tamura K: **MEGA X: Molecular Evolutionary Genetics Analysis across Computing Platforms**. *Mol Biol Evol* 2018, **35**(6):1547-1549.
5. Seibold PS, Lenz C, Gressler M, Hoffmeister D: **The *Laetiporus* polyketide synthase LpaA produces a series of antifungal polyenes**. *J Antibiot (Tokyo)* 2020, **73**(10):711-720.
6. Geib E, Brock M: **ATNT: an enhanced system for expression of polycistronic secondary metabolite gene clusters in *Aspergillus niger***. *Fungal Biol Biotechnol* 2017, **4**:13.
7. Lawrinowitz S, Wurlitzer JM, Weiss D, Arndt HD, Kothe E, Gressler M, Hoffmeister D: **Blue Light-Dependent Pre-mRNA Splicing Controls Pigment Biosynthesis in the Mushroom *Terana caerulea***. *Microbiol Spectr* 2022, **10**(5):e0106522.
